# Overexpression of synaptic vesicle protein Rab GTPase 3C promotes vesicular exocytosis and drug resistance in colorectal cancer cells

**DOI:** 10.1002/1878-0261.13378

**Published:** 2023-02-14

**Authors:** Yu‐Chan Chang, Chien‐Hsiu Li, Ming‐Hsien Chan, Chih‐Yeu Fang, Zhi‐Xuan Zhang, Chi‐Long Chen, Michael Hsiao

**Affiliations:** ^1^ Department of Biomedical Imaging and Radiological Sciences National Yang Ming Chiao Tung University Taipei Taiwan; ^2^ Genomics Research Center Academia Sinica Taipei Taiwan; ^3^ National Institute of Infectious Diseases and Vaccinology National Health Research Institutes Miaoli Taiwan; ^4^ Department of Pathology, Taipei Medical University Hospital and College of Medicine Taipei Medical University Taiwan; ^5^ Department and Graduate Institute of Veterinary Medicine, School of Veterinary Medicine National Taiwan University Taipei Taiwan

**Keywords:** cannabinoid receptor type 2, colorectal cancer, drug resistance, dystrophin, Rab GTPase 3C

## Abstract

Rab GTPase 3C (RAB3C) is a peripheral membrane protein that is involved in membrane trafficking (vesicle formation) and cell movement. Recently, researchers have noted the exocytosis of RAB proteins, and their dysregulation is correlated with drug resistance and the altered tumor microenvironment in tumorigenesis. However, the molecular mechanisms of exocytotic RABs in the carcinogenicity of colorectal cancer (CRC) remain unknown. Researchers have used various *in silico* datasets to evaluate the expression profiles of RAB family members. We confirmed that RAB3C plays a key role in CRC progression. Its overexpression promotes exocytosis and is related to the resistance to several chemotherapeutic drugs. We established a proteomic dataset based on RAB3C, and found that dystrophin is one of the proteins that is upregulated with the overexpression of *RAB3C*. According to our results, RAB3C‐induced dystrophin expression promotes vesicle formation and packaging. A connectivity map predicted that the cannabinoid receptor 2 (CB2) agonists reverse RAB3C‐associated drug resistance, and that these agonists have synergistic effects when combined with standard chemotherapy regimens. Moreover, we found high dystrophin expression levels in CRC patients with poor survival outcomes. A combination of the dystrophin and *RAB3C* expression profiles can serve as an independent prognostic factor in CRC and is associated with several clinicopathological parameters. In addition, the RAB3C–dystrophin axis is positively correlated with the phosphatidylinositol 4,5‐bisphosphate 3‐kinase catalytic subunit alpha isoform (*PIK3CA*) genetic alterations in CRC patients. These findings can be used to provide novel combined therapeutic options for the treatment of CRC.

## Graphical abstract

The authors found that *RAB3C* overexpression induces dystrophin expression to promote vesicle formation and packaging for increased exocytosis, is related to drug resistance and is correlated with PIK3CA/KRAS mutation status in colon cancer. RAB3C‐based omics profiles identified inhibitors that directly interact with dystrophin and found synergy with existing chemotherapy regimens.

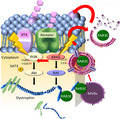

**Abbreviations**
5‐FU: 5‐fluouracil
APC: adenomatous polyposis coli
Cav‐1: caveolin‐1
CB2: cannabinoid receptor type 2
CIMP: CpG island methylator phenotype
EMT: epithelial‐mesenchymal transition
EV: extracellular vesicle
GPCR: G protein‐coupled receptor
IHC: immunohistochemistry
ILV: intraluminal vesicle
IPI: International Protein Index
KRAS: Kirsten rat sarcoma viral proto‐oncogene
LINCS: library of integrated network‐based cellular signatures
LTQ: linear ion trap
LTQ‐FT LC/MS/MS: LTQ tandem mass spectrometer
MS: microsatellite
MVB: multivesicular body
NTA: nanoparticle tracking analysis
PIK3CA: phosphatidylinositol‐4,5‐bisphosphate 3‐kinase
RAB: Ras‐related protein
RAB3C: Rab GTPase 3C
TCGA: The Cancer Genome Atlas
TEM: transmission electron microspore
TMA: tissue microarray

## 1 Introduction

Colorectal cancer (CRC) is considered one of the highest‐risk cancers in the world [1]. Although chemotherapy and a combination of surgery and chemotherapy can successfully control some of the disease progression, drug resistance and cancer metastasis remain severe issues and are the primary causes of the fatalities in CRC. The tumorigenesis of CRC consists of several genetic mutations and epigenetic modification events. Researchers have investigated the genetic alteration events of adenomatous polyposis coli (APC) [2], phosphatidylinositol‐4,5‐bisphosphate 3‐kinase (PIK3CA) [3, 4, 5], Kirsten rat sarcoma viral proto‐oncogene (KRAS) [6], and the tumor protein p53 (TP53) [7], and they have found that these events can induce multiple individual pathways or increase the mutation loads of clinical patients. In addition, the CpG island methylator phenotype (CIMP) status [8, 9] and microsatellite (MS) status [10, 11] also affect the treatment strategies. Moreover, most patients are diagnosed at an advanced stage, which limits the therapeutic options for achieving good responses and leads to a poor prognosis. Therefore, we are searching for the key factors that are associated with the drug resistance and cancer metastasis in CRC to resolve this dilemma and improve the outcomes of patients.

Recently, researchers identified the Ras‐related protein (RAB) small GTPase family as a key family that regulates membrane trafficking [12, 13, 14], exosome formation [15, 16], and even pathway transduction [17]. The RAB3 family participates in the activation–inactivation cascade of RAB26, RAB27, and RAB37, which causes exocytosis [15, 16, 18, 19]. The process of exocytosis involves cytokine/chemokine secretion, drug efflux, and autocrine pathway activation [20, 21]. In our previous study, we revealed that RAB3C is upregulated in CRC and interacts with other family members [22]. Similarly, compared with normal adjacent tissues, we also observed the increased expression of RAB3C in tumor tissues. We have established RAB3C‐based transcriptomic datasets and provided evidence that RAB3C promotes cancer metastasis through the IL‐6/STAT3 axis to affect patient survival [22]. Nevertheless, we note that RAB3C plays an additional role in the mechanisms that rely on exocytosis. Therefore, in the current study we evaluated the increase in the vesicle formation (exosomes, multivesicular bodies, and lysosomes) in an RAB3C‐overexpression model. We demonstrated that RAB3C is the most important prognostic factor among the RAB3 family members. RAB3C enhances the exocytosis of CRC cells and increases the resistance of several typical chemotherapy drugs (5‐FU, oxaliplatin, and regorafenib) [23, 24, 25].

Exosomes, or EVs, are small membrane vesicles of endocytic origin that contain mRNAs, DNA fragments, and proteins, and that are released by many different cell types, including cancer cells. Tumor‐derived exosomes are involved in the formation and progression of different cancer processes, including tumor microenvironment remodeling, angiogenesis, metastasis, and drug resistance [26]. Researchers have shown interest in exosome‐derived vectors for tracking, delivery, and therapy. However, there are still many unknown concepts as to their detailed contents and mechanisms of action. The RAB3 family complex that consists of RAB3C is involved in exosome trafficking and its translocation [27]. We speculate that the overexpression of RAB3C may lead to the formation and secretion of abundant exosomes in CRC. Researchers have identified several chemical drugs in exosomes, which may also be the main cause of the chemoresistance [28].

We demonstrated that dystrophin is one of the abundant proteins that is overexpressed in our RAB3C‐based proteomic dataset from three independent overexpression cell models. Indeed, we demonstrated the direct binding of RAB3C to dystrophin through protein–protein interactions and their regulation of the drug resistance in CRC cells. We also demonstrated that the signature of the combined expression profiles of RAB3C and dystrophin could be an independent prognostic factor for CRC patients through immunohistochemistry staining. Most important, this event was associated with frequent PIK3CA/KRAS mutations in patients with CRC. We hypothesized that certain drugs or compounds might reverse these phenotypes by interfering with the RAB3C–dystrophin interaction and thereby enhancing the chemosensitivity. Therefore, we utilized a connectivity map to seek potential candidates. According to our results, in CRC the treatment of the cannabinoid receptor (CB2) agonist could synergize with conventional chemotherapeutic agents to inhibit colon cancer cells. Taken together, we propose a novel strategy for the clinical application of the CB2 agonist for the enhancement of the chemotherapeutic drug response in colorectal patients with high expressions of RAB3C.

## 2 Materials and methods

### 2.1 Cell lines and stable clones

We cultured human colorectal SW48 (RRID:CVCL_1724), SW480 (RRID:CVCL_0546), and SW620 (RRID:CVCL_0547) cancer cells in L‐15 medium and Dulbecco's modified Eagle medium (DMEM). We cultured the colorectal HT‐29 (RRID:CVCL_A8EZ) and HCT116 (RRID:CVCL_0291) cancer cells in McCoy's 5A medium, and the CX‐1 (RRID:CVCL_2011), DLD‐1 (RRID:CVCL_0248), and H3347 (RRID:CVCL_LG07) CRC cells in RPMI medium that was supplemented with 10% fetal bovine serum (FBS). We incubated all the cells under a humidified atmosphere of 5% CO_2_ at 37 °C. We purchased SW48, SW480, SW620, HCT116, and DLD‐1 from the ATCC cell bank. These authenticated cell lines were received within the last 3 years with a certificate. CX‐1 and H3347 were gifts from Prof Wei‐Shone Chen (Division of Colon & Rectal Surgery, Department of Surgery, Taipei Veterans General Hospital, Taipei, Taiwan). CX‐1 and H3347 cells were authenticated by short tandem repeat (STR) analysis, yielding more than an 80% match in profiled loci. For this study, all cell lines were identified as mycoplasma‐free using an assay kit.

### 2.2 Gene construction and lentivirus production

We purchased the lentiviral envelope and packing plasmid (pMDG and p▵8.91) from the National RNAi Core Facility (Academia Sinica, Taiwan). We purchased the plenti6.3–RAB3C lentiviral constructs and empty vectors from Addgene (Watertown, MA, USA). We cotransfected the lentiviruses into 293T cells with pMDG, p▵8.91, and the plasmid construct using the calcium phosphate transfection method. After 48 h of incubation, we collected the lentiviruses and used them to infect the cells with polybrene (2 μg·mL^−1^). We selected the cells with altered RAB3C expressions with blasticidin (2 μg·mL^−1^) for 2 weeks. We used a plasmid carrying a vector control sequence to create the control cells. The detailed process and classification are introduced in our previous articles [22].

### 2.3 CRC sample selection and immunohistochemical analysis

In total, 215 patients diagnosed with colorectal adenocarcinoma at the Taipei Municipal Wan Fang Hospital of Taiwan from 1998 to 2005 were included in this study. We retrieved the CRC tissues from the Department of Pathology, Taipei Municipal Wan Fang Hospital (Taipei, Taiwan), with Institutional Review Board approval. All experiments were approved by the Ethical Committee (Taipei Medical University‐Joint IRB, approval number: TMU‐IRB 99049). All methodologies conformed to the standards set by the Declaration of Helsinki. Informed consent was signed by all patients and all experiments were approved by the Ethical Committee. We fixed the surgical specimens in 10% buffered neutral formalin and embedded them in paraffin. We reviewed the histological diagnoses, tumor sizes, levels of tumor invasiveness, and lymph node statuses of all the cases, and two pathologists (M.H. and C.L.C.) confirmed them. We determined the final disease stages according to the Cancer Staging System of the American Joint Committee of Cancer (AJCC). We retrospectively collected the clinical data, including data on the follow‐up period, overall survival period, and disease‐free survival period, from each patient's medical record. We followed the patients for more than 152 months or until their deaths. We excluded the patients who died of postoperative complications within 30 days of the surgery from the survival analysis [29].

We used a tissue microarray (TMA) for the immunohistochemistry (IHC) analysis of the RAB3C expression in this study [22]. We prepared the TMA containing the CRC tissues and corresponding adjacent noncancerous colon tissues, as previously described [22]. For each case, we selected three 1‐mm cores from different areas of the tumor tissue. In addition, if available, we also selected two 1‐mm cores of adjacent noncancerous normal colon mucosa for each case. In total, we assembled 243 archival CRC samples for the TMA. The antibodies that we used for the IHC staining included antihuman RAB3C (1:100; Cat # 15029‐1‐AP, Proteintech, Rosemont, IL, USA) and dystrophin (1:50; Cat # HPA023885, Atlas Antibodies, Bromma, Sweden). We performed the immunodetection with an EnVision dual‐link‐system horseradish peroxidase (HRP) detection kit (DAKO, Glostrup, Denmark). The detailed process and classification were introduced in our previous articles [22].

### 2.4 Immunohistochemical scoring

We devised a four‐point staining‐intensity scoring system to determine the RAB3C/dystrophin expression in the CRC TMA specimens, and the staining intensity scores ranged from 0 (no expression) to 3 (high expression). We classified the results into two groups according to the intensity and extent of the staining: in the low‐expression group; either no staining was present (staining intensity score = 0), or we detected positive staining in fewer than 10% of the cells (staining intensity score = 1); in the high‐expression group, positive immunostaining was present in 10–30% of the cells (staining intensity score = 2), or in more than 30% (staining intensity score = 3). The extent of staining was scored by the percentage of positive cells (0–100%). The final IHC scores (0–300) were obtained by staining intensity score multiplied by the percentage of positive cells. All cases were divided into two groups according to the final IHC scores and processed 50% cutoff. Low IHC expression level was defined as a score less than 150 and a score more than 150 included and 150 itself was defined as high expression. Two pathologists reviewed and independently scored the IHC staining results [30]. The detailed process and classification are introduced in our previous articles [22].

### 2.5 Animal studies

All animal experiments were conducted in accordance with the Guide for the Use and Care of Laboratory Animals (Animal Research: Reporting of *In Vivo* Experiments guidelines), and the animal protocol (AS IACUC No. 19‐12‐1398) was approved by the Experimental Animal Committee, Academia Sinica, Taiwan. We used age‐matched severe combined immunodeficient gamma (JAXTM NOD.Cg‐Prkdcscid Il2rgtm1Wjl/SzJ; NOD‐SCIDγ) male mice at 6 weeks old from Jackson Laboratory (Bar Harbor, ME, USA). Animals were housed in a sterile environment in cages with autoclaved bedding, food, and water and maintained on a daily 12 h light, 12 h dark cycle. In addition, we randomly divided the 18 mice into three groups (*n* = 6 per cage) for further experiments. For the estimation of the *in vivo* tumorigenicity, we resuspended 5 × 10^6^ colon cancer cells in 100 μL of phosphate‐buffered saline (PBS) and subcutaneously injected them under the dorsal skins of the mice. When the subcutaneous tumor size reached 0.5 cm, we initiated the different treatments: the sham group received the PBS treatment. We used the Vernier caliper measurement of two perpendicular tumor diameters (*L* and *W*) to monitor the tumor growth once a week. We calculated the tumor volume using the formula *LW* ^2^/2. We measured the bodyweights weekly. We stopped administering the treatment to the animals in the regorafenib group when the bodyweights decreased to below 80% of the starting bodyweights. We harvested the tumor masses after 6 weeks of treatment. We euthanized the mice when the tumor volume reached 1500 mm^3^, or in case of severe weight loss or necrosis, with CO_2_ in the air chamber. We maintained the CO_2_ flow until the animals stopped breathing. The detailed process and classification are introduced in our previous articles [22]. The Institutional Ethics Committees approved the study protocol, and the animal experimental design was approved by the animal facility committee (AAALAC/ICLAS) under the approved procedure.

### 2.6 Cell viability measurements

We determined the cell viability using the TACS tetrazolium salt 3‐(4,5‐dimethylthiazol‐2‐yl)‐2, 5‐diphenyltetrazolium bromide (MTT) cell proliferation assay kit (Trevigen, Gaithersburg, MD, USA), according to the manufacturer's instructions. Researchers use MTT to determine the cell viability in cell proliferation and cytotoxicity assays. We seeded the cells at a concentration of 2000 cells per 100 μL culture media per well into 96‐well microplates. At 24 h postseeding, we treated the cells with the dimethyl sulfoxide (DMSO) solvent control or different doses of regorafenib for 24, 48, or 72 h. Subsequently, we incubated the cells in a medium containing MTT for 4 h, lysed them by DMSO, and we then measured the optical density at 570 nm using a microplate reader (Spectral Max250; Molecular Devices, Sunnyvale, CA, USA). The detailed process and classification were introduced in our previous articles [30].

### 2.7 Isolation and purification of exosomes secreted by donor cells

For the separation of the exosomes from the conditioned medium of Cx‐1, DLD‐1, and Hct116 cells, we harvested 160 mL of RPMI and Mccoy5A serum‐free medium after conducting it in 15‐cm dishes, and we then removed the cells and cell debris from the conditioned medium by centrifugation at 500 × ***g*** for 10 min and 2000 × ***g*** for 30 min. We further filtered the supernatant through a 0.22‐μm filter and ultrahigh speed centrifugation at 100 000 × ***g*** (36 900 r.p.m.) for 90 min (Beckman 70Ti rotor; Brea, CA, USA). We resuspended the pellets in 20 mm 4‐(2‐hydroxyethyl)‐1‐piperazineethanesulfonic acid (HEPES), and we loaded them on top of the sucrose gradients with the indicated compositions (from bottom to top: 1.2 mL of 2 m; 2 mL of 1.3 m; 2 mL of 1.16 m; 1.8 mL of 0.8 m; 1.8 mL of 0.5 m; 1.2 mL of 0.25 m, with 20 mm of Tris at a pH of 7.4). We performed the ultracentrifugation at 100 000 × ***g*** (28 500 r.p.m.) for 16 h (Beckman SW41 rotor), and we collected 0.5 mL from each fraction. We washed the isolated EVs 3 times with PBS through Amicon 0.5 mL centrifugal filters (100 kDa; Millipore, Billerica, MA, USA) for concentration and further analysis [31]. We recruited several relevant molecules to validate each fraction (Fig. S1).

### 2.8 Electron microscopy and multivesicular body (MVB) quantification

We washed the cells cultured on dishes in PBS and fixed them for 1 h in 2.5% glutaraldehyde in 0.1 m phosphate buffer at room temperature. Then we slowly and gently scraped and pelleted the cells in Eppendorf tubes. We washed the pellets in phosphate buffer and incubated them with 1% OsO_4_ for 90 min at 4 °C. Then we dehydrated the samples, embedded them in Spurr, and sectioned them using a Leica ultramicrotome (Leica Microsystems, Vista, CA, USA) [32, 33]. We stained ultrathin sections (50–70 nm) with 2% uranyl acetate for 10 min, and with a lead‐staining solution for 5 min, and we observed them using a transmission electron microscope (Hitachi TEM system, Tokyo, Japan). We used imagej for the calibration, quantification, and analysis of the images (National Institutes of Health, Bethesda, MD, USA). We identified the MVBs and counted them by morphology, having only discrete ILVs. The lysosomes revealed the multilayer morphology. We analyzed at least 20 MVBs per experiment from the separate cells. We analyzed the data from duplicate or triplicate experiments, and we used from two to four grids for each condition. The minimum number of cells scored for each condition was 20. We generated the box scatterplots using prism graphpad software (GraphPad Software, Boston, MA, USA), and we performed the statistical tests in Microsoft Excel (calibration: 2.0; magnification: × 2.0 k–× 6.0 k; lens mode: Zoom‐1; acc. voltage: 75.0 kV; emission: 5.8 μA). Data are means ± standard deviations; ***P‐value < 0.0001.

### 2.9 Nanoparticle tracking analysis

We used the nanoparticle tracking analysis (NTA) system to analyze the particle size distribution in the exosome sample. The system is equipped with a 488 nm laser and a high‐sensitivity scientific CMOS camera, and it was used with the NanoSight NS300 system (Malvern Technologies, Malvern, UK). According to the manufacturer's specifications, we diluted the sample at 1:100 in particle‐free PBS to an acceptable recommended concentration to reduce the number of particles in the field of view to below 100/frame. We took the reading videos in a single capture during 60 s at 30 frames per second (fps), with the camera level set to 15 and manual temperature monitoring. Then we used the nta 3.1.54 software (Malvern Panalytical Ltd, Malvern, UK) to divide the particle size distribution into 10‐nm wide intervals, which is used for all the video reproduction to determine the concentration measurement value. To understand the variability in the estimated value within the entire interval width, the software organizes the statistical data obtained by each frame.

### 2.10 Exosome membrane labeling

Researchers commonly use fluorescent dyes to label the cellular membrane for exosome labeling because the lipid bilayers in exosomes are a good target. Here, we chose the ExoParkler Exosome Membrane Labeling Lit‐Red (Dojindo, Kumamoto, Japan), which enabled us to use the application to experiment using multiple labels. After collecting 10^9^–10^10^ exosomes suspended in 100 μL PBS, we added 2 μL Mem Dye stock solution and mixed it with the exosomes. We transferred the staining exosomes to a filtration tube and centrifuged them at 3000 × ***g*** for 5 min about three times. We added 50 μL of PBS to recover the labeled exosomes.

### 2.11 L1000 and LINCS analysis

L1000 is an innovative gene expression profiling technique with a high‐throughput scale (20 × 384 samples per week) for next‐generation pharmaceutical discovery applications. By using L1000 mining, we could predict the potential compounds that inhibit our input event (RAB3C overexpression). We can generate genetic perturbations using dedicated pattern‐matching algorithms in the Library of Integrated Network‐based Cellular Signatures (LINCS; https://lincsproject.org/). The LINCS is an innovative gene expression profiling solution for next‐generation pharmaceutical discovery applications. It is a high‐throughput (20 × 384 sample per week) and low‐cost (~15% of regular array costs) gene expression profiling platform that was built at the Broad Institute (Cambridge, MA, USA) [34, 35]. By using L1000 profiling, we can access the expression data generated from a large collection of small molecules through a Google‐like search engine, which allows us to connect disease indications with potential lead compounds by dedicated pattern‐matching algorithms. The LINCS dataset includes 3000 human genes, including the known targets of FDA‐approved drugs, drug–target pathway members, and candidate disease genes, which researchers have perturbed using lentivirally delivered shRNAs in the same set of 15 cell lines [36]. We list the gene perturbagen candidates in Table S1, which we generated from a query of the regorafenib treatment gene signature in the LINCS. We then prioritized the perturbagen candidates by their connectivity scores across the four cell lines, in which the perturbagen gene signature was most strongly connected to that of the regorafenib treatment and cutoff at a connectivity score of ≧ 90.

### 2.12 Western blot analysis

We lysed the cells in RIPA buffer for 30 min, and we then centrifugated them at 13 000 r.p.m. for 15 min at 4 °C. We obtained the membrane/cytoplasmic protein fractions of the cultured cells with the Mem‐PER Plus Membrane Protein Extraction kit (Thermo, Waltham, MA, USA). We measured the protein concentration using BCA protein assay reagents (Thermo). We separated the total proteins (30 μg) by SDS–PAGE on 10% polyacrylamide gels, and we transferred them to a PVDF membrane. We hybridized the membranes with primary antibodies overnight after blocking for 30 min in 5% nonfat milk. We incubated the samples with the secondary antibodies for 1 h, and we then visualized the proteins using enhanced chemiluminescence (ECL) reagents (Perkin Elmer, Waltham, MA, USA). We obtained the quantitative data using imagej software. The detailed process and classification are introduced in our previous articles [22].

### 2.13 In‐gel digestion and LC–MS/MS analysis

We stained the gel with Coomassie Blue (J.T. Baker, Radnor, PA, USA) for 10 min, as described above for SDS/PAGE, and we then cut it into strips and washed it once with ddH_2_O. We destained the gel pieces with 50% acetonitrile (ACN)/25 mm ammonium bicarbonate overnight at 4 °C. We stored each part of the gel in ddH_2_O at 4 °C. We discarded the gel containing liquid and replaced it with reduction buffer (10 mm DTT/25 mm ABC) for 1 h at 56 °C. The reduction buffer and added alkylation buffer (55 mm IAA/25 mm ABC) were in darkness for 1 h at room temperature. We discarded the solution and washed it twice with 40% ACN for 10 min, and we then dehydrated it by treatment with 100% ACN. We dried the gel pieces under vacuum and rewet them with 0.12 μg of modified trypsin (Promega, Madison, WI, USA) in 25 mm ABC, and we then digested them overnight at 37 °C. We transferred the solutions of the peptides to new Eppendorf tubes and extracted them using two 100‐μL portions of 60% ACN/0.1% trifluoroacetic acid (TFA). We dried the solution in vacuum, and we redissolved the peptides in 0.1% TFA for the LC–MS/MS analyses [37].

We performed the LC–MS/MS analyses on a linear ion trap (LTQ) tandem mass spectrometer (LTQ‐FT LC/MS/MS, Thermo Electron). We used the mascot search engine (Matrix Science, Plano, TX, USA) for the protein identification, according to a curated protein database (International Protein Index [IPI] human database, compiled by the European Bioinformatics Institute). We established the peptide mass tolerance and fragment mass tolerance at 100 ppm and 0.25 Da, respectively. We scored the proteins using a probability‐based MOWSE algorithm (for MOlecular Weight SEarch), and we reported the mascot scores in the form of −10 × log(*P*), where *P* is the probability that the observed match is a random event. We searched the acquired data against the Human Protein Sequence Database of the International Protein Index (IPI) by using the automated database‐searching program mascot (Matrix Science). We searched the spectra with mass tolerances of 15 ppm for the MS data, and with mass tolerances of 0.8 Da for the MS/MS data. We allowed up to two missed trypsin cleavages. We set the carbamidomethyl cysteine as the fixed modification, and we set the oxidized methionine and deamidation as the variable modifications. We summarized and exported all the identified proteins as a spreadsheet in.xlsx file format.

### 2.14 The treatment of exosomes (cocultured assays)

We seeded 10^5^–10^6^ Cx‐1 cells on a 6‐cm dish (#3295; Corning, Corning, NY, USA) and incubated them overnight. The next day, we rinsed the dish twice with PBS to remove the residual RPMI and replaced it with serum‐free RPMI. After adding about 10^9^–10^10^ exosomes (50 μL of the labeled exosomes), we incubated them for 2 days for the subsequent analysis. To facilitate the exosome uptake by the recipient cells, we reduced the volume of the culture medium to half of the normal volume [22].

### 2.15 Immunoprecipitation and immunoblotting analyses

We incubated the whole‐cell lysates (2 mg) from the cultured cells overnight in IP buffer with 25 μL of protein A/G magnetic beads and the corresponding antibodies against RAB3C (Cat # 15029‐1‐AP, Proteintech, Rosemont, IL, USA) or dystrophin (Cat # HPA023885, Atlas antibodies, Bromma, Sweden) in a 1.5‐mL microcentrifuge tube with a final volume of 1000 μL. We purified the proteins that interacted with the antibodies according to the manufacturer's protocol [38].

### 2.16 Migration assay

For the migration assays, we coated 8‐μm‐pore‐size polycarbonate filters (GE Healthcare Life Sciences, Chalfont St. Giles, UK) with 1 mg·mL^−1^ human fibronectin (Sigma, St. Louis, MO, USA). We added a medium containing 10% fetal bovine serum (FBS) to the lower compartment, and we added cells suspended in a serum‐free medium to the upper compartment of the Boyden chamber. After the optimized timing (12–16 h), we stained the migrating cells with the Giemsa solution and counted them under a light microscope (400×, eight random fields of each well) for further quantification. We performed three independent experiments with four replicates each. The detailed process and classification are introduced in our previous articles [30].

### 2.17 Synergy calculation

We formulated all the dosing groups in a dose‐dependent manner from 1 nm to 1 μm to calculate the cell viability percentages. We uploaded these values to the SynergyFinder website for the statistical calculations (https://synergyfinder.fimm.fi/). We present the synergy scores and degrees of inhibition for the two compounds based on the available algorithms (synergy score (δ‐score) < 10: antagonistic; −10 ~ 10: additive; > 10: synergistic).

### 2.18 Statistical analysis

The nonparametric Mann–Whitney *U*‐test was used to analyze the statistical significance of results from three independent experiments. Statistical analyses were performed using spss (Statistical Package for the Social Sciences) 17.0 software (SPSS, Chicago, IL, USA). A paired *t*‐test was performed to compare the RAB3C/dystrophin IHC expression levels in cancer tissues and in the corresponding normal adjacent tissues. Pearson's chi‐square test analyzed the association between clinicopathological categorical variables and the RAB3C/dystrophin IHC expression levels. Estimates of the survival rates were calculated using the KM method and compared using the log‐rank test. Follow‐up time was censored if the patient was lost during follow‐up. Univariate and multivariate analyses were performed using Cox proportional hazards regression analysis with and without an adjustment for RAB3C/dystrophin IHC expression level, tumor stage, lymph node stage, and metastasis. For all analyses, a *P*‐value of < 0.05 was considered significant.

We list the detailed resources and conditions in Table S2.

## 3 Results

### 3.1 RAB3C regulates exosome formation and vesicle trafficking

**Fig. 1:**
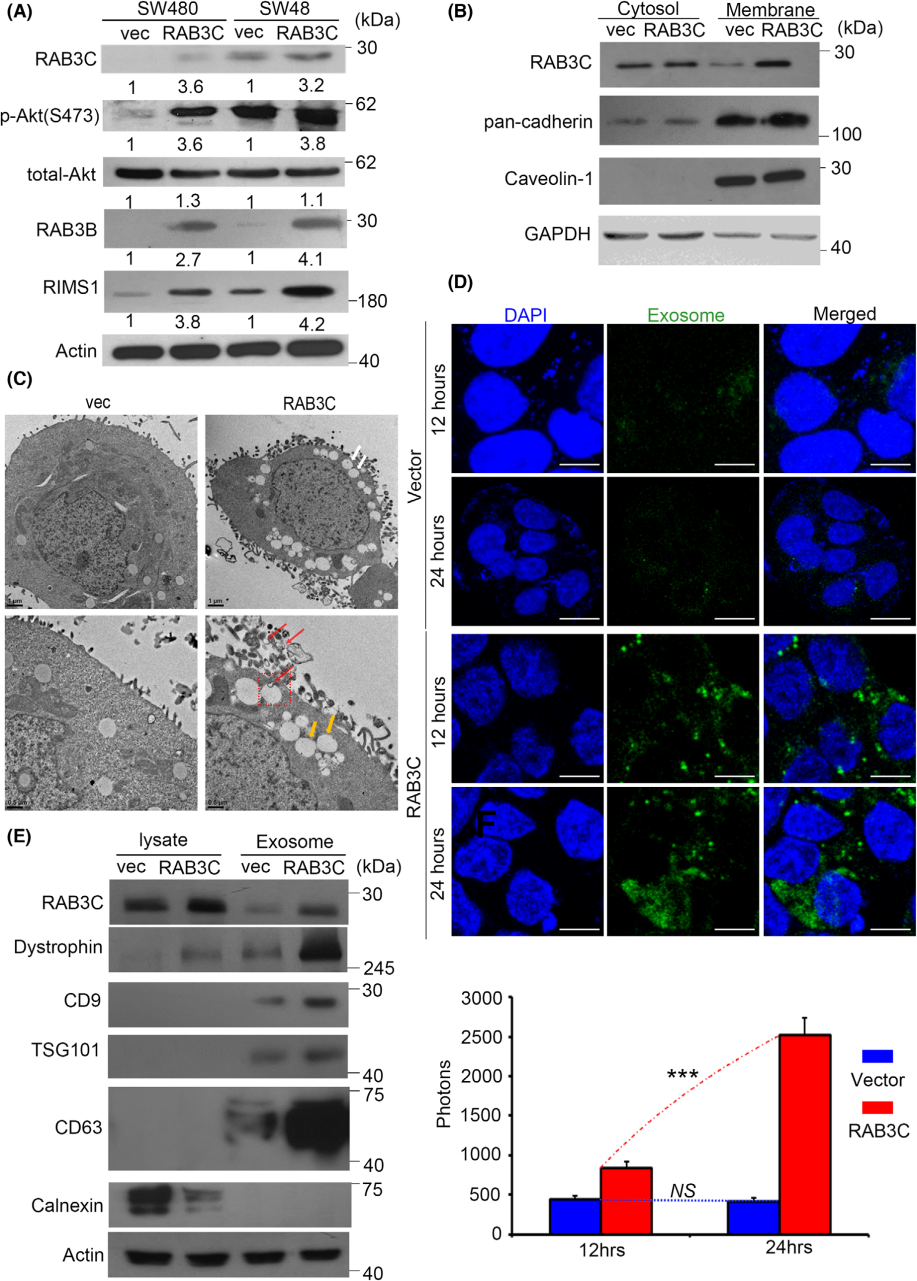
RAB3C regulates signaling transduction, vesicle formation, and exocytosis. (A) Western blots showing the RAB3C, total‐/phosphor‐Akt, RAB3B, and RIMS1 in the RAB3C‐overexpression models. (B) Intracellular expression of RAB3C in CRC cells after definition of cytoplasm/cell membrane. (C) Representative images of RAB3C‐expression model in SW480 cells examined by transmission electron microscopy (TEM). White arrows indicate exocy.tosis; yellow and red arrows indicate ILVs and MVBs, respectively. ILVs: intraluminal vesicles; MVBs: multivesicular bodies. Scale bar: 1 and 0.5 μm, respectively. Red arrows indicate exosomes. (D) (upper) Representative images and (lower) quantification of exosomes between vector and RAB3C‐overexpression models in SW480 cells examined by confocal microscopy. Analysis represents exosomal membranes. Blue: DAPI. Green: exosome membrane. Scale bar: 25 μm. (E) Western blot analysis of expressions of RAB3C, calnexin, dystrophin, CD9, CD63, and TSG101 of whole cells and exosomes isolated in RAB3C‐expression models. ***P < 0.001, NS, not significant. Data are presented as the mean ± standard error of the mean. Student's *t*‐test was used for the comparison of measurable variants of two groups. All experiments were performed with at least three biological duplicates (*n* = 3) for each group, in triplicate.

RAB GTPase is involved in many aspects of membrane trafficking, including vesicle formation, movement, and fusion and cytoskeleton modeling. RABs are involved in processes that involve endosomes, exosomes, autophagosomes, lysosomes, etc. RAB3C can promote CRC cell migration/invasion through the autocrine regulatory signaling pathways, and it can serve as a prognostic indicator in CRC patients [22]. However, whether RAB3C itself regulates exocytosis is still unclear. In addition, we found that conditioned supernatant has a similar tendency to promote certain cancer phenotypes. Therefore, we propose that RAB3C may be involved in the regulation of the exocytosis of CRC cells and the formation of exosomes (Fig. S1). We analyzed the RAB3C protein levels in normal colonic epithelial cells (CoEpics). According to our results, RAB3C was substantially more expressed in the tumor cells than in normal primary cells (Fig. S2). We further established RAB3C‐overexpression cell models in several low‐endogenous‐expression colon cancer cells, including SW480 and SW48. In previous studies, researchers have claimed that exosomes derived from cancer cells promote the proliferation of recipient cells via the PI3K/Akt pathway. The phosphorylation of Akt should be a pivotal event of exocytosis [39]. After confirming the overexpression of RAB3C, we observed an increase in the phosphorylation status of Akt (Ser 473). At the same time, the exosome‐related RAB3B and the exocytosis marker RIMS1 were also increased (Fig. 1A). We can also obtain complementary results if we perform knockdown experiments using shRNA (Fig. S3). According to previous reports, RAB3C is involved in exocytosis (moving from the cytoplasm to the cell membrane), the fusion into multivesicular bodies (MVBs) and exosomes, and colocalization with various other Rabs [40]. Meanwhile, some scaffold molecules, such as caveolin‐1 (Cav‐1) or cadherins, participate in the vesicle formation and secretion that sublocalizes to the cell membrane. Cells that contain Cav‐1 can also use exosomes as carriers to remove intracellular Cav‐1 and secrete them as vesicle cargo [41]. Therefore, we used membrane and cytoplasmic fractions to observe whether RAB3C promotes this mechanism. Our results confirmed that RAB3C and Cav‐1 were translocated to the membrane, presumably already preparing for exocytosis via early endosomes and MVBs (Fig. 1B). Therefore, we tried to determine the importance of RAB3C in exocytosis. To confirm that the overexpression of RAB3C can promote exocytosis and vesicle trafficking, we dissected the formation state and used transmission electron microscopy (TEM) to verify that there was more vesicle formation in the RAB3C‐overexpression group (Fig. 1C). From this vesicle formation in cells, we can presume the intraluminal vesicles (ILVs) and multivesicular bodies (MVBs). We also tagged the exosomes with green fluorescent protein (GFP), and we found that the RAB3C group secreted and produced more substantial amounts (Fig. 1D). We based the quantification on the florescent signal (Fig. 1D and Fig. S4), and according to the results, more exosomes were formed in the RAB3C group, which is consistent with the use of purified exosomes, which has also been characterized by NTA (particle size confirmation) to detect the extracellular vesicle flux (Videos S1 and S2 and Fig. S5). According to Fig. S5, the exosome size did not differ much before or after the overexpression of RAB3C (control: 118 nm; RAB3C overexpression: 97 nm). Conversely, the RAB3C knockdown model also showed complementary trends (Videos [Link], [Link] and Fig. S6). Furthermore, we purified the exosomes and examined the molecular markers associated with the exosomes and our targets, including RAB3C, dystrophin, CD9, TSG101, CD63, and calnexin (Fig. 1E). According to the combined evidence, RAB3C plays an important role in the preparation, flux, and efficiency of exocytosis.

### 3.2 RAB3C coordinates with dystrophin in colorectal cancer to promote various phenotypes

**Fig. 2:**
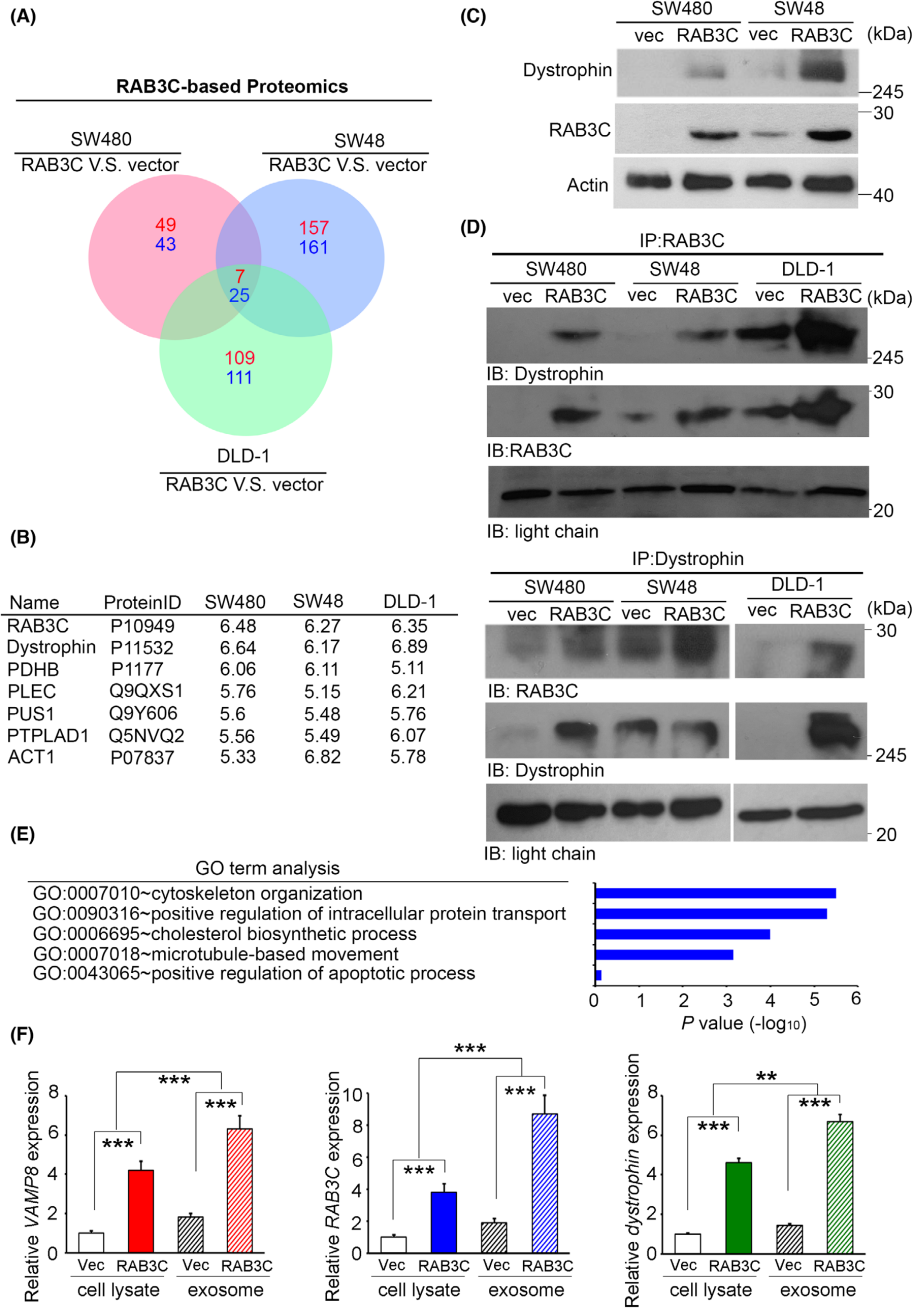
RAB3C/dystrophin complex modulates exosome formation, vesicle trafficking, and drug resistance. (A) Venn diagrams of common interacting molecules in proteomic profiles of SW480, SW48, and DLD‐1 RAB3C models. (B) List of and fold‐changes in common interacting molecules in RAB3C‐overexpression models. (C) Western blot analysis of RAB3C and dystrophin protein expressions in SW480 and SW48 cells with or without RAB3C overexpression. (D) We performed RAB3C/dystrophin‐related immunoprecipitation assays on whole‐cell lysates from SW480‐, SW48‐, and DLD‐1‐ RAB3C‐overexpression models. (E) List of biological functions for RAB3C‐interacting molecules by gene annotation analysis. (F) qRT‐PCR analysis of *VAMP8*, *RAB3C*, and *dystrophin* expressions of whole cells and exosomes isolated in RAB3C‐expression models. **P < 0.01, ***P < 0.001. Data are presented as the mean ± standard error of the mean. Student's *t*‐test was used for the comparison of measurable variants of two groups. All experiments (C/D/F) were performed with at least three biological duplicates (*n* = 3) for each group, in triplicate.

To confirm whether there are other molecules involved in the vesicle formation, fusion, or translocation during RAB3C‐overexpression‐induced exocytosis, we established the proteomic profiles of several RAB3C‐overexpression models by comparing the alterations in the protein expressions in parental and RAB3C‐overexpressing cells. After the trypsin digestion and mass spectrometry analysis of the protein extracts from these cells, we identified the potential targets with >5‐fold changes in all three RAB3C‐based cell models through a Venn diagram analysis (Fig. 2A). These commonly altered molecules include seven standard upregulators and 25 standard downregulators (Fig. 2B and Table S3). Among them, dystrophin has the most substantial and consistent trend in all three cell models. We validated each of these proteins in RAB3C‐overexpression mode; however, some behaved inconsistently with their proteomic signatures (Fig. S7A). In addition, we focused on the positive correlation between the candidate and RAB3C expression levels in the TCGA_CRC cohort (Fig. S7B,C). We then focused on dystrophin, and we found that its protein level was profoundly increased in the RAB3C‐expression model (Fig. 2C). Based on previous reports, dystrophin is involved in cytoskeleton rearrangement and exosome packing during exocytosis [42]. The loss of the dystrophin function in cancer cells contributes to cell movement or metastasis. Therefore, we performed a two‐way immunoprecipitation assay to confirm the interaction status between RAB3C and dystrophin, and we observed the valid binding of RAB3C and dystrophin in all three overexpression models (Fig. 2D and Fig. S8). Meanwhile, according to the gene annotation analysis, the organization of the cytoskeleton, cellular transport, and microtubule‐related trafficking are the most affected features in RAB3C‐overexpressing cells (Fig. 2E). In order to investigate the protein–protein‐interaction state, we further processed the protease inhibitor MG‐132, and we observed that dystrophin behaved differently from the vector in the RAB3C model (Fig. S9). This evidence may also indicate that the cytoskeleton reorganizes during exocytosis, and that dystrophin reassembles membrane components and actin filaments and promotes exocytosis [43, 44]. Therefore, we isolated exosomes from the RAB3C‐overexpression cells to evaluate whether the RAB3C and dystrophin were translocated and packed into them. According to our data, the vesicle‐associated proteins VAMP8, dystrophin, and RAB3C were all presented in the exosomes, and their expressions were increased in the RAB3C‐overexpression model (Fig. 2F). When we attempted to suppress the dystrophin expression in an RAB3C‐overexpression model, several exosome‐related markers also decreased (Fig. S10). According to these results, dystrophin contributes to RAB3C‐induced exocytosis.

### 3.3 Synergistic effect of CB2 agonist AM1241 and chemotherapy drugs in RAB3C‐overexpressing models

**Fig. 3:**
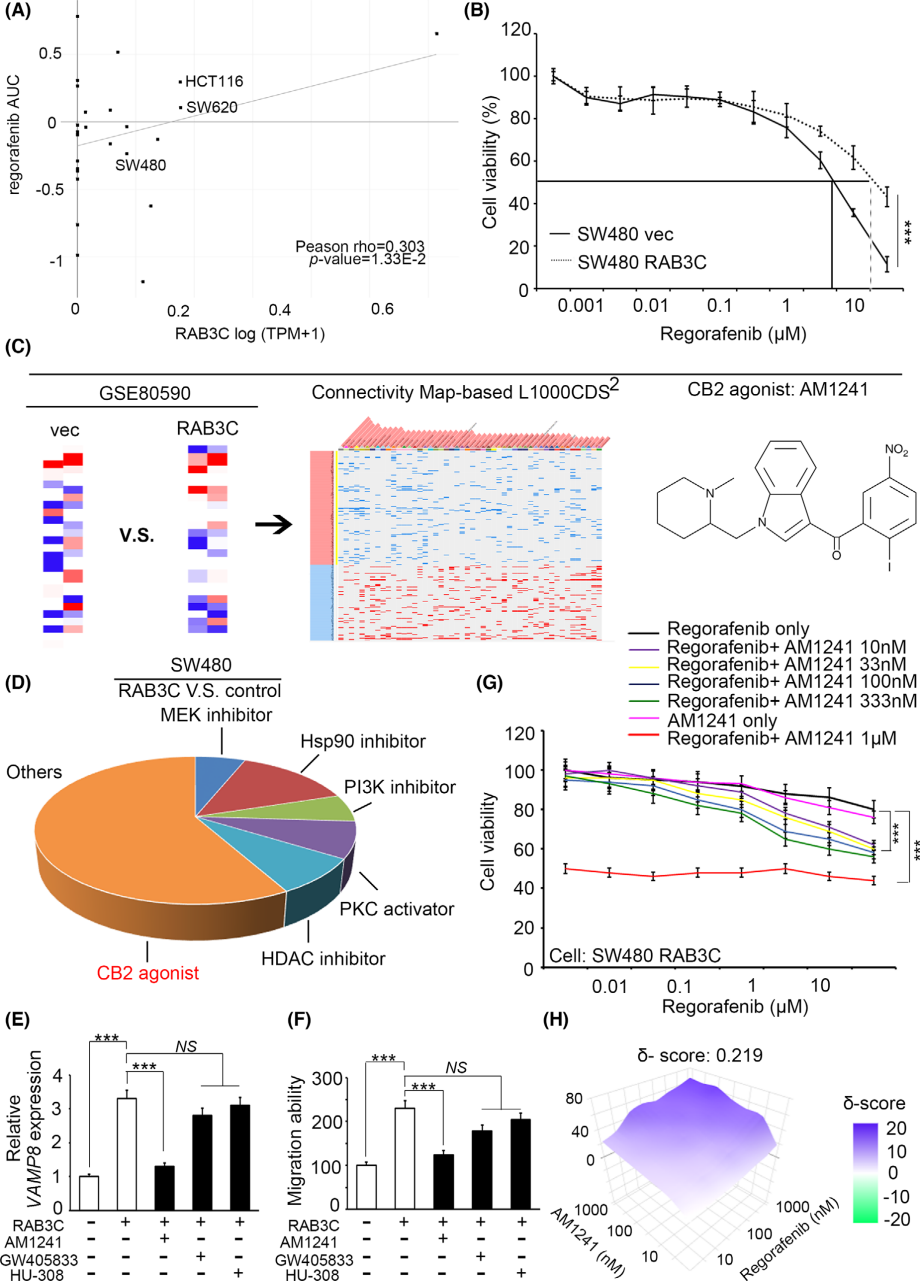
Synergy of regorafenib and CB2 agonists in the RAB3C overexpression model of CRC. (A) Correlation plot showing the relationship between RAB3C‐expression level (Expression 21Q4 public) and the IC50 of regorafenib (BRD:BRD‐K16730910‐001‐10‐7) from the DepMap portal website. Pearson *r* = 0.303; *P*‐value = 0.0133. (B) Cell viability of regorafenib treatment in RAB3C‐expression model in SW480 cells. (C) Flowchart of candidate drug screening and prediction through integrated signatures of transcriptomics from the Connectivity website (https://maayanlab.cloud/L1000CDS2/#/index). (D) Potential compound candidates predicted by SW480 RAB3C‐overexpression transcriptomic profiles from the L1000 website. (E) qRT‐PCR analysis of *VAMP8* with or without treatment with CB2 agonists in RAB3C‐expression model. (F) Detection of migration ability with or without treatment with CB2 agonists in the RAB3C‐expression model. (G) Cell viability under treatment of regorafenib alone or combined with various dosages of CB2 agonist AM1241 (10 nm–1 μm) in the SW480 RAB3C‐overexpression model. (H) Synergy calculation of combined use of regorafnib and AM1241 through SynergyFinder service (synergy score (δ‐score) < 10: antagonistic; −10 ~ 10: additive; > 10: synergistic). ***P < 0.001, NS, not significant. Data are presented as the mean ± standard error of the mean. Student's *t*‐test was used for the comparison of measurable variants of two groups. All experiments (B/E) were performed with at least three biological duplicates (*n* = 3) for each group, in triplicate.

We next assessed the association of RAB3C‐regulated exosomes with migration, invasion, and a series of EMT‐related events in CRC cell (Fig. S11). Moreover, we assessed whether the viability of standard chemotherapy for CRC is affected by RAB3C overexpression. Exosomes have been implicated in their drug resistance, and researchers have also demonstrated their regulation through the PTEN/Akt pathway [39, 45], which is consistent with our observations (Fig. 1A). Thus, we screened several chemotherapeutic agents, including regorafenib (an antiangiogenic agent that is commonly used for the treatment of metastatic CRC), oxaliplatin, and 5‐FU. According to the results, there were consistent trends across the multiple chemodrugs; however, the regorafenib sensitivity was inversely correlated with the RAB3C‐expression level in colon cancer (Fig. 3A and Fig. S12). Mimicking the clinical situation, 5‐FU and oxaliplatin are now primarily used in combination, rather than alone. Therefore, we chose regorafenib as the control group, and we examined the treatment response in the RAB3C‐overexpression model. According to our results, the high RAB3C expression resulted in an increased resistance to regorafenib (Fig. 3B).

To overcome the drug resistance effect and seek ways to enhance the drug sensitivity in RAB3C‐expressing tumors, we assumed that the candidate compounds could reverse the phenotypes induced by RAB3C overexpression. We selected about 1000 probes that were altered in the RAB3C‐expression groups with more than a 1.5‐fold change. Through the L1000 CDS2 website (Characteristic Direction Signature Search Engine), we found that PKC activators, PI3K inhibitors, MEK inhibitors, Hsp90 inhibitors, and HDAC inhibitors were predicted to have the reverse effect of RAB3C overexpression (Fig. 3C and Table S1). However, some of the prediction options above did not yield significant results (Fig. S13). The cannabinoid receptor type 2 (CB2) agonist was also considered to be an RAB3C‐induced phenotypic inhibitor in colon cancer cells (Fig. 3D and Fig. S13). Researchers have reported a cannabinoid that inhibits the cancer‐derived extracellular vesicle release in a dose‐dependent manner [46]. We recruited several CB2 agonists, including GW405833, AM1241, and HU‐308. Our findings suggest that AM1241 more substantially inhibits the phenotypes than the other drugs (Fig. 3E,F). We then investigated the antiproliferative effect of the combination of the CB2 agonist and regorafenib. According to the results, the combined treatment of AM1241 and regorafenib had a better effect at inhibiting the viability of the colon cancer cells (Fig. 3G and Fig. S14), and after calculating the multiplicative effect, the combination indeed had a synergistic effect (Fig. 3H).

**Fig. 4:**
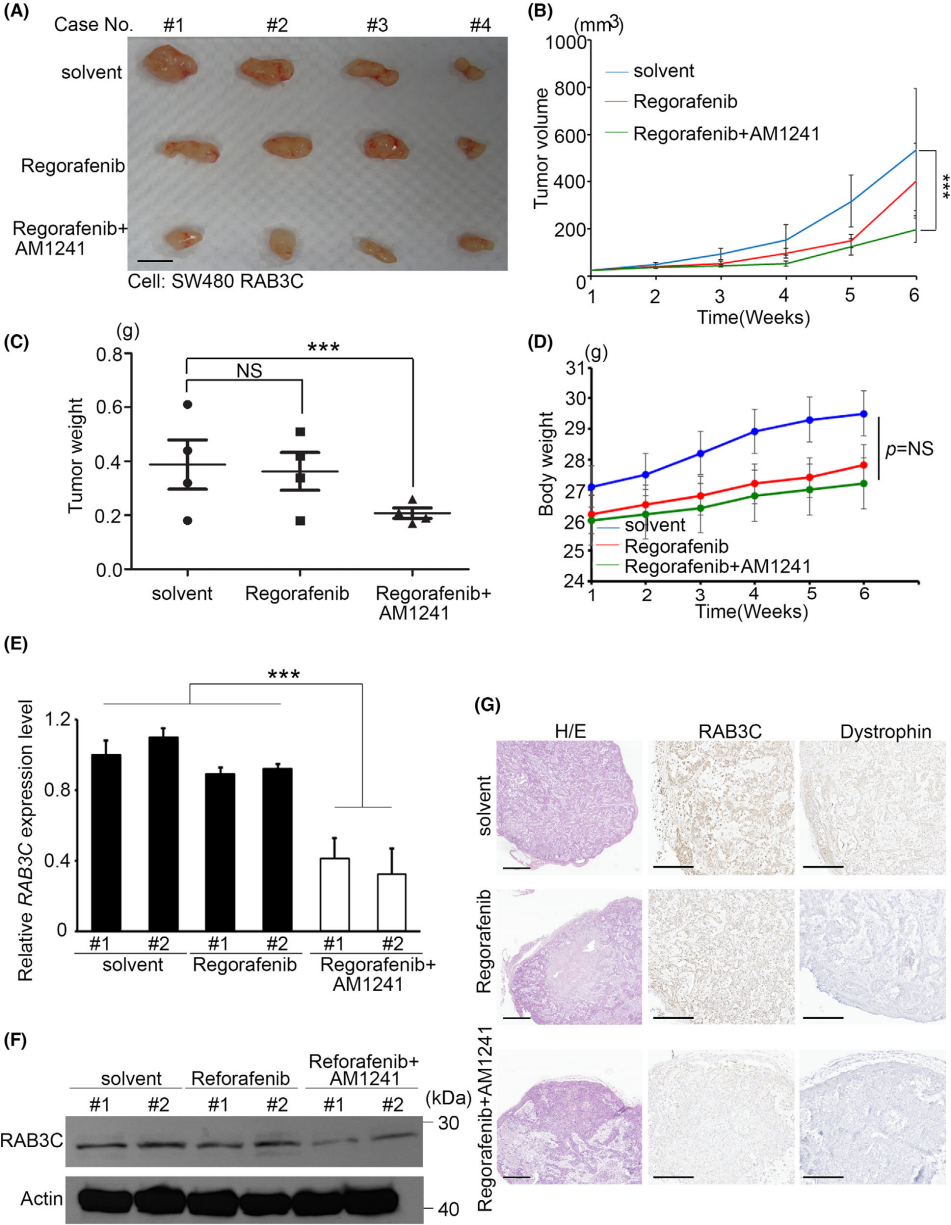
Synergistic effect of regorafenib and CB2 agonists *in vivo*. (A) Overview of solid tumor response to regorafenib alone or regorafenib combined with AM1241 compared with control group *in vivo*. Scale bar: 1 cm. (B) Tumor growth curve of solvent control, regorafenib alone, and regorafenib combined with AM1241 *in vivo*. (C) Quantitation of tumor weights in mice with solvent, regorafenib alone, and regorafenib combined with AM1241 treatments (*P* = 0.019). (D) Bodyweights of mice treated with solvent, regorafenib alone, and regorafenib combined with AM1241 *in vivo*. (E) qRT‐PCR analysis of *RAB3C* in solid tumors of mice treated with solvent, regorafenib alone, and regorafenib combined with AM1241. (F) Western blot analysis of RAB3C protein expression in solid tumors of mice treated with solvent, regorafenib alone, and regorafenib combined with AM1241. We used regorafenib at a concentration of 10 μm in this study, and at a concentration of 1 μm for AM1241. (G) Representative images of dystrophin/RAB3C protein staining and H/E staining results in xenograft models. We used paired *t*‐tests to analyze statistical significance in control and regorafenib groups. Scale bar: 500 μm. ***P < 0.001, NS, not significant. Data are presented as the mean ± standard error of the mean. Student's *t*‐test was used for the comparison of measurable variants of two groups. All experiments were performed with at least three biological duplicates (*n* = 3) for each group, in triplicate.

We validated this combination effect in mouse models by the subcutaneous injection of colon cancer cells, followed by the drug treatments. According to our data, the combination of regorafenib and AM1241 had the most substantial effect on the tumor growth inhibition *in vivo* compared with the solvent control (Fig. 4A). Furthermore, we found that the tumor volume can only be inhibited by combination therapy, while regorafenib alone had no significant effect *in vivo* (Fig. 4B,C). In particular, the bodyweights of each group were not reduced by regorafenib or combination therapy (Fig. 4D). To determine the consistent efficiency of the combination, we also examined the RAB3C expression using mouse xenograft tumors, which showed that the RNA and protein levels were reduced in the *in vivo* model (Fig. 4E,F). According to the results of the hematoxylin and eosin (H/E) and immunohistochemical staining of the xenograft model, the combination group inhibited the tumor growth and expressions of RAB3C and dystrophin (Fig. 4G), which suggests that CB2 agonists may provide an additional treatment option for patients with colon cancer, and that they have a synergistic effect in combination with regorafenib.

### 3.4 The synergistic effect of activated RAB3C and dystrophin in CRC is associated with clinicopathological events

**Fig. 5:**
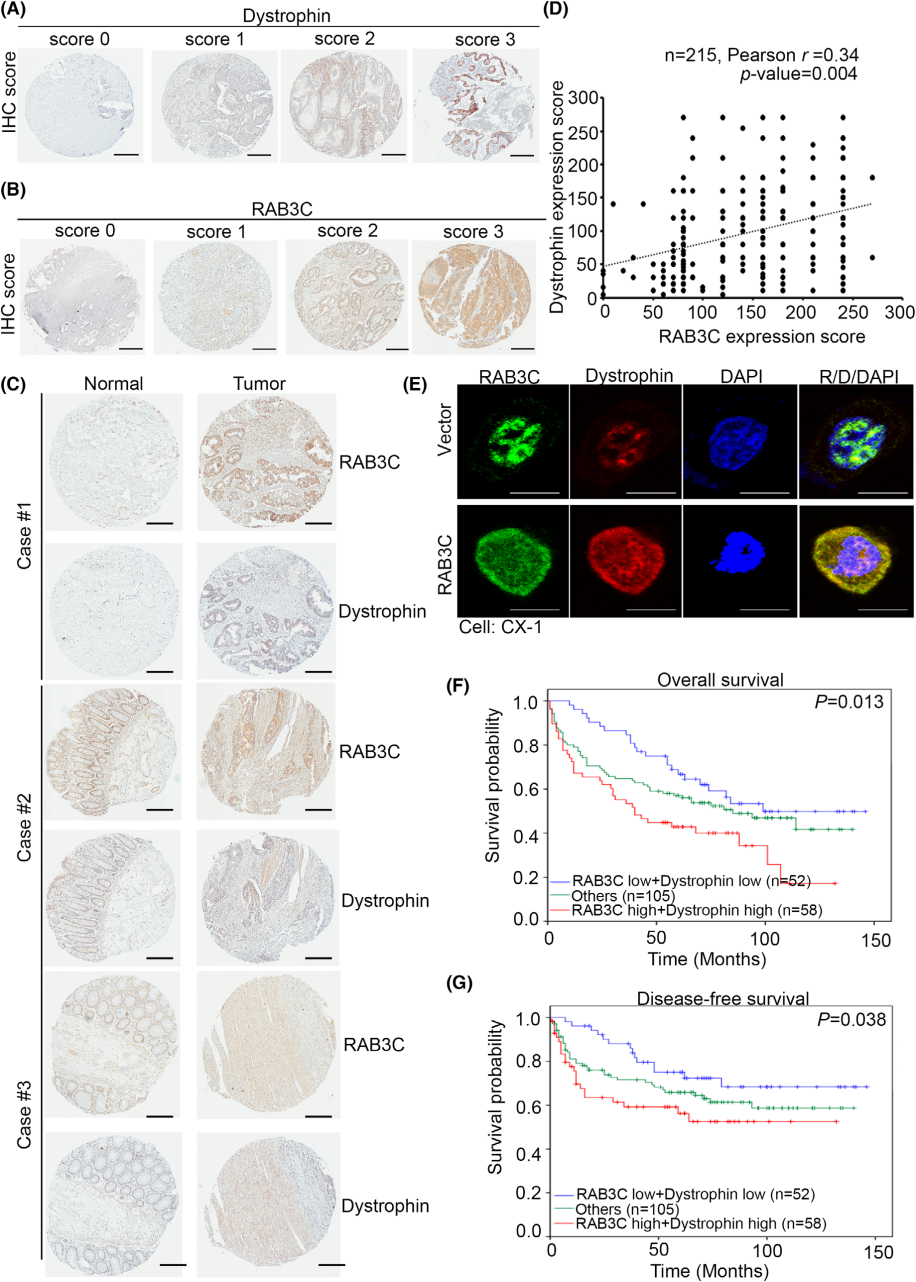
Combined RAB3C and dystrophin serve as an independent indicator of poor prognosis, distant metastasis, and advanced stage in CRC patients. (A) Scores indicating dystrophin levels in representative colorectal tumor tissues from 0 to 3. Scale bar: 300 μm. (B) Scores indicating RAB3C levels in representative colorectal tumor tissues from 0 to 3. Scale bar: 300 μm. (C) Representative image of dystrophin/RAB3C‐protein‐staining results in normal adjacent tissue/tumor pairs of patients. Scale bar: 300 μm. (D) Correlation statistics between RAB3C and dystrophin protein in CRC tissue microarray (*n* = 215; Pearson's *r* = 0.34; *P* = 0.004). (E) Representative fluorescent images of dystrophin/RAB3C‐protein‐staining results in RAB3C‐expression model in CX‐1 cells. Scale bar: 10 μm. (F) Kaplan–Meier curves of overall and (G) disease‐free survival of 215 patients with CRC, stratified by high or low RAB3C, combined with dystrophin protein expression levels (*P* = 0.013 and *P* = 0.038, respectively). “Others” include RAB3C high + dystrophin low group and RAB3C low + dystrophin high group. All experiments were performed with at least three biological duplicates (*n* = 3) for each group, in triplicate.

To determine whether the RAB3C and dystrophin are related to the clinicopathological parameters of cancer patients, we performed the IHC staining of dystrophin and RAB3C on the colorectal tissue arrays to assess whether their expressions can be considered predictors of a poor prognosis in CRC (Fig. 5A,B). According to our data, the RAB3C and dystrophin demonstrated coordinated expression, and the tumor tissues exhibited stronger staining than the adjacent normal tissues (Fig. 5C,D). According to the cell experiments, RAB3C and dystrophin have colocalization, both exist in the nucleus and cytoplasm, and their overexpression increases the distribution on the cell membrane (Fig. 5E and Fig. S15). Furthermore, according to the Kaplan–Meier survival analysis, the combination of RAB3C and dystrophin was a significant predictor of the patient survival and could be further used to categorize patients into different risk groups in terms of both overall survival (Fig. 5F) and disease‐free survival (Fig. 5G). The univariate and multivariate analyses also demonstrated the independent prognostic value of RAB3C combined with dystrophin in colon cancer patients (Table S4). According to these data, the RAB3C/dystrophin protein has prognostic value in cancer progression, and it can be used as a therapeutic target for improving the survival rates of patients.

### 3.5 The RAB3C protein expression level was positively correlated with genetic events of PIK3CA and KRAS

**Fig. 6:**
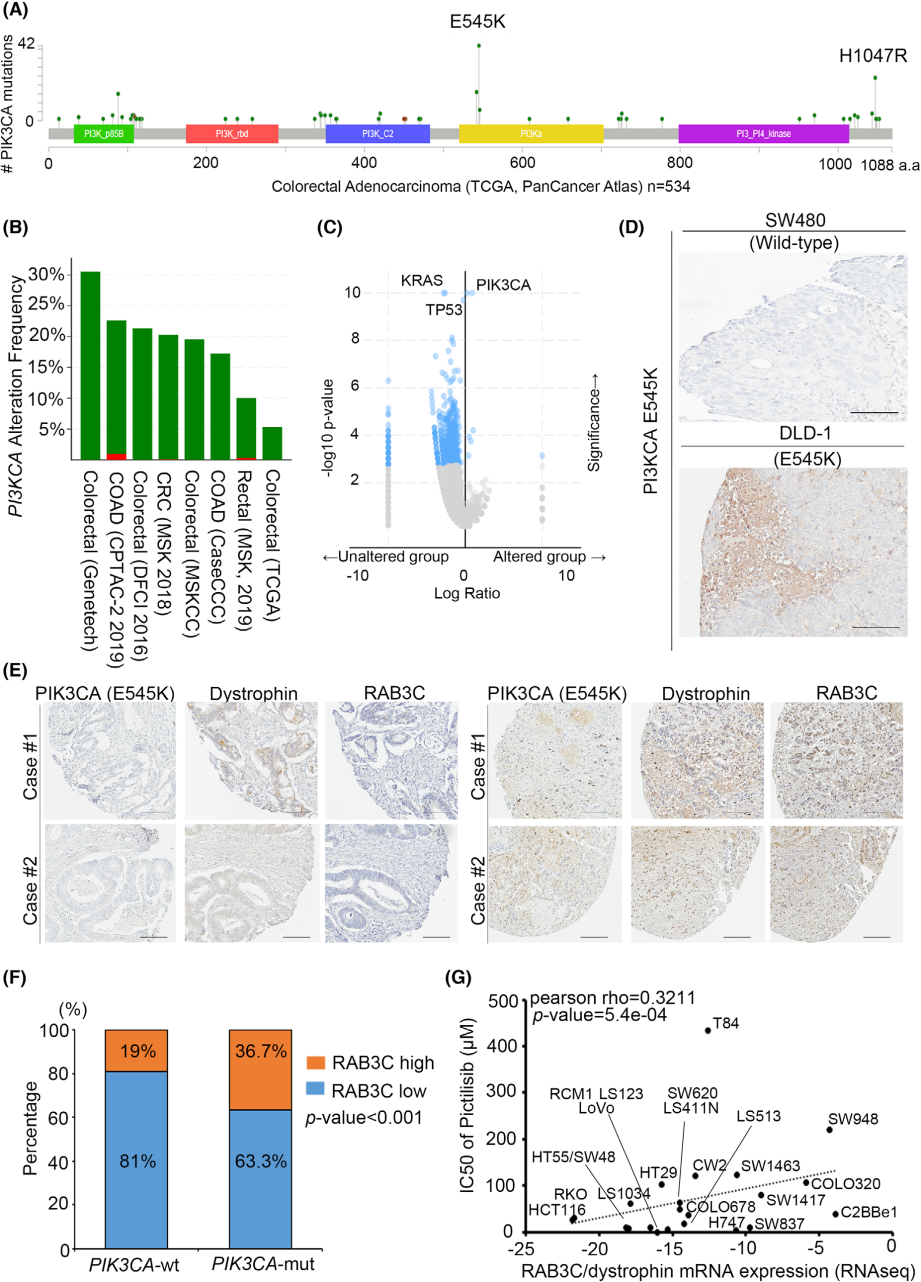
RAB3C expression level is correlated with genetic events of *PIK3CA/KRAS* in CRC patients. (A) Protein domains of *PIK3CA* and its mutation sites in colorectal adenocarcinoma patients from TCGA clinical cohort (*n* = 534) from GEPIA. (B) Bar graph showing frequency of *PIK3CA* alterations in various clinical cohorts. Green: gene mutation. Red: amplification. (C) Ranking of genetic modification events in colon cancer cohorts. (D) Representative image of PIK3CA E545K mutant protein staining results in colon cancer cells. (E) Representative image of dystrophin/RAB3C/PIK3CA E545‐protein‐staining results in clinical specimens. (F) Percentage of RAB3C‐expression levels in *PIK3CA* wildtype and mutant groups, respectively (wt: *PIK3CA* gene is wildtype; mut: *PIK3CA* gene mutation). (G) Correlation plot of IC50 of pictilisib with RAB3C/dystrophin mRNA expression levels in colon cancer cell lines. Scale bar: 300 μm. All experiments were performed with at least three biological duplicates (*n* = 3) for each group, in triplicate.

**Fig. 7:**
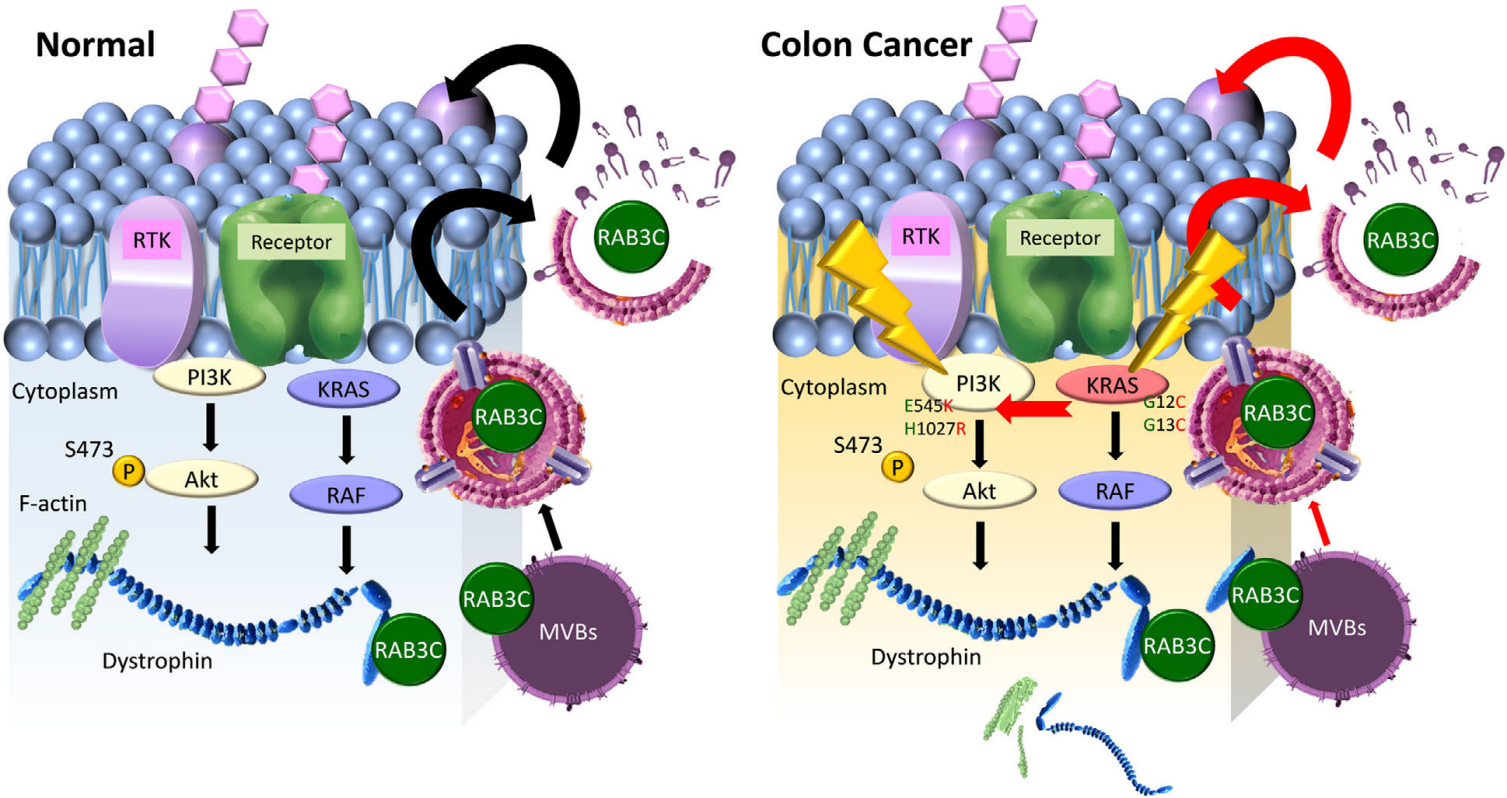
Schematic representation of the study. This illustration integrates our evidence for the ability of RAB3C to regulate the exosome formation, its interaction with dystrophin, and its relevance to gene‐altering events in CRC.

Mutations in the *PIK3CA* or *KRAS* genes are considered to be one of the main genetic variants of CRC. More important, these genetic alterations are constitutively activated oncogenic signals for proliferation and exocytosis [47]. We therefore dissected the proposal that aberrant RAB3C expression is associated with genetic mutations in the recruited cell lines and clinical samples. We analyzed the mutation hotspots of *PIK3CA* and *KRAS* in several CRC cohorts on the GEPIA website (Fig. 6A and Fig. S16A). We observed that 10~30% of colorectal adenocarcinomas are associated with *PIK3CA* mutations, while 20~50% of patients are associated with a *KRAS* mutation status (Fig. 6B and Fig. S16B). Via the ranking of the genetic modification events in the colon cancer cohorts, *KRAS*, *PIK3CA*, and *TP53* are the most important targets in colon cancer patients (Fig. 6C). In addition, we confirmed that two *PIK3CA* mutation events (E545K and H1047R) are the most common mutant forms of the *PIK3CA* gene (Fig. S16C). Therefore, we performed an IHC analysis of the *PIK3CA*‐specific mutant forms for the genetic alteration events in CRC cell lines. According to our data, we detected mutant forms of *PIK3CA* expression in DLD‐1 (E545K), while we did not detect any in SW480 (wildtype; Fig. 6D). We also examined the endogenous RAB3C‐expression level in the CRC cell panel, which could respond to the PIK3CA genetic alteration events (Fig. S16D). Using the PIK3CA E545K mutant antibody expression classification, dystrophin and RAB3C were overexpressed in the positive group and underexpressed in the negative group (Fig. 6E). Based on these criteria, the clinical cases had substantial differences (Fig. 6F). Researchers have reported that KRAS alteration events can promote the poor survival of clinical patients (Fig. S17); however, there was no significant correlation with our results. Following a similar procedure, we also included APC and β‐catenin (CTNNB1) mutation events for the analysis [48]. After calculation, the RAB3C of the APC and β‐catenin expressions were not different between the mutation and wildtype groups (Fig. S18). To identify potential strategies for further application in the clinic, we compared the IC_50_ of several PIK3CA inhibitors and the endogenous levels of RAB3C and dystrophin in the colon cancer cell panel. According to our results, high RAB3C/dystrophin expression may lead to PIK3CA inhibitor resistance, including pictilisib, NVPBEZ235, and AZD6482 (Fig. 6G and Fig. S19). According to these results, in *PI3KCA* mutation cases, the expression level and interaction state of RAB3C/dystrophin are influential. Thus, detecting the RAB3C/dystrophin expression in colon cancer can reflect the patient's prognosis, as well as his/her response to targeted therapy applications (Fig. 7).

## 4 Discussion

Researchers have identified RAB3C as an integral part of exosomes, and they have shown its interactions with other family members, including RAB3A/B/D, RAB26, RAB27, and RAB37 [49, 51]. However, in this study we observed that RAB3C translocates and regulates the exosomal vesicles, and it forms protein–protein interactions during tumorigenesis. Therefore, we established an RAB3C‐based proteomic approach, and we found that several factors are implicated in RAB3C‐related pathogenesis. Apart from RAB3C and dystrophin, pyruvate dehydrogenase E1 beta subunit (PDHB), plectin (PLEC), pseudouridine synthase 1 (PUS1), 3‐hydroxyacyl‐coa dehydratase 3 (HACD3, PTPLAD1), and TRAF3 interacting protein 2 (TRAF3IP2, ACT1) were also upregulated in the three RAB3C‐overexpressing cell models. Nonetheless, we prioritized dystrophin as our target to coordinate the upregulation in CRC through a competitive statistical analysis. Here we demonstrate that RAB3C dominates the drug resistance and exocytosis in colon cancer, and that dystrophin also plays a role. Both are overexpressed in tumorigenesis, and RAB3C stabilizes dystrophin. According to previous studies, dystrophin is a cohesive protein that links actin filaments (F‐actin) with another supportive protein to the main cell cytoskeleton [50]. During exocytosis, F‐actin polymerizes to the periphery to facilitate vesicle docking and fusion, which, in turn, allows membrane indentation, budding, and exocytosis [52, 53]. We confirmed the exact interaction site and validated the binding site with small sequence peptides. We established the crystal structure of RAB3C. We evaluated which small compounds can block the binding affinity and have strong binding energies and stabilization abilities through the virtual screening system. Other molecules are worthy of investigation as prognostic factors or detailed mechanisms in the future.

Recently, researchers have reported that secretory RABs control the exosome secretion in cancer cells, which functions in the facilitation of angiogenesis, degrading the extracellular matrix and creating an immune‐privileged environment for cancer cells [54, 55, 56]. Researchers have isolated the cancer progression markers, including the molecules for metastasis and signaling transduction, as well as some lipid‐raft‐associated proteins, from metastatic colon cancer‐derived exosomes [57, 58]. Additionally, they have also reported the level of circulating exosomes to be an indicator for colon cancer prognosis [59, 60]. Exosomes also affect the chemoresistance and chemosensitivity of cancer cells through the drug efflux mechanisms of cytotoxic drugs, such as cisplatin and taxanes [61, 62]. Furthermore, several studies on the inhibition of exosome liberation through interference with exocytotic RABs have also provided new insights in the study of the chemoresistance mechanisms.

As a standard protocol of CRC treatment, 5‐FU‐based therapeutic agents are the primary regimen for treating patients. Many chemotherapeutic regimens are currently being adapted for the treatment of CRC. The conventional treatment of advanced CRC encompasses a combination of 5‐FU and leucovorin with oxaliplatin or irinotecan (FOLFOXIRI) [23, 24]. Regorafenib has been used as a second‐line drug for treating CRC [25, 63]. Despite the advancement of different therapeutic protocols for the treatment of CRC, this cancer still displays specific mechanisms that result in a lower therapeutic benefit, and especially in advanced and recurrent tumors. In this study, we determined that the interaction of RAB3C and dystrophin may lead to chemoresistance in CRC, which may provide a new therapeutic target for overcoming the drug resistance in this disease.

The *PIK3CA* mutant status is one of the hallmarks of CRC. Approximately 30% of clinical patients have shown *PIK3CA* mutants through next‐generation sequencing (NGS) analysis [64, 65]. In response to this condition, researchers developed alpelisib and fulvestrant as PIK3 inhibitors; however, they are still in clinical trials [66]. The KRAS inhibitor sotorasib is currently in a phase II clinical trial in CRC and a phase III trial in nonsmall‐cell lung cancer patients [67]. Additional therapeutic strategies are urgently needed for CRC patients with frequent mutational events. We predicted the candidate compounds using RAB3C‐based gene signatures from the Connectivity‐MAP database. This also means that RAB3C and its interactomes have higher performance/similarity, and that inhibitors can have a dramatic effect. From this result, we speculate that AM1241 can have an effect on RAB3C and render the available drug effective. A combination with the available chemotherapy approaches could result in a synergistic effect for clinical patients. In follow‐up studies, researchers should also focus on whether AM1241 can prevent exocytosis and the RAB3C/dystrophin interaction, and compare it with the other predicted inhibitors. Moreover, we observed that RAB3C/dystrophin proteins are prevalent in PI3K mutant cells. In summary, we propose that CB2 agonists may have synergistic effects with standard chemotherapy in CRC. In our next study, we aim to determine the interaction affinity between RAB3C and dystrophin in *PIK3CA* or *KRAS* mutant events. Moreover, whether the RAB3C/dystrophin gene level and exocytosis ability changes are different under genetic alterations is also a direction that needs to be studied.

## 5 Conclusions

In this study we screened the expression levels of RAB GTPases, and we found that RAB3C is the most important factor for vesicle formation, drug resistance, and clinical events in CRC. According to the RAB3C‐based proteomic datasets, dystrophin is one of the critical molecules in RAB3C‐based proteomics, and it directly binds RAB3C. For drug repurposing, CB2 agonists have been selected for synergy with chemotherapy drugs. The binding of CB2 agonists causes conformational changes in the G‐protein‐coupled receptors (GPCRs) that facilitate the coupling to intracellular proteins and initiates signaling cascades. Researchers have studied some Rabs that involve the slow recovery of GPCRs [68]. We suggest that CB2 agonists may abolish this change. In addition, CB2 agonists have antiinflammatory effects [69], which also echoes our previous findings that RAB3C cultivates the IL‐6/STAT3 axis in colon tumorigenesis [22]. These combined approaches can reverse the malignant phenotype *in vitro* and *in vivo*. Moreover, the combination profile of RAB3C and dystrophin is an independent prognostic factor for CRC patients (Fig. 7).

## Conflict of interest

The authors declare no conflict of interest.

## Author contributions

Conception and design: C‐LC, MH. Development of methodology: Y‐CC, M‐HC, C‐HL, C‐YF, Z‐XZ. Acquisition of data (provided animals, acquired and managed patients, provided facilities, etc.): Y‐CC, M‐HC, C‐HL, C‐YF, Z‐XZ. Analysis and interpretation of data (e.g., statistical analysis, biostatistics, computational analysis): Y‐CC, C‐YF, C‐LC, MH. Writing, review, and/or revision of the article: Y‐CC, M‐HC, C‐HL, C‐YF, C‐LC, MH. Administrative, technical, or material support (i.e., reporting or organizing data, constructing databases): C‐LC, MH. Study supervision: MH.

### Peer review

The peer review history for this article is available at https://publons.com/publon/10.1002/1878‐0261.13378.

## Supporting information


**Table S1.** Small compound candidates predicted by L1000 CDS^2^ in SW480 RAB3C cells.
**Table S2.** Detailed resources and reagents used in this study.
**Table S3.** List of common signatures in RAB3C‐based proteomics. Normalized to the vector control group and ordered by Log ratio.
**Table S4.** Univariate and multivariate analyses for RAB3C/dystrophin expression in colorectal cancer.
**Fig. S1.** Fraction collection after sucrose gradient separation and examination of exosome‐related markers.
**Fig. S2.** Western blot analysis of RAB3C protein expression in various colon cancer cells and normal colon epithelial cells.
**Fig. S3.** Western blots showed the RAB3C, total‐/phosphor‐Akt, RAB3B and RIMS1 in RAB3C knockdown models.
**Fig. S4.** ROI region delineation and quantification of each group of confocal images.
**Fig. S5.** Exosome concentration (particles·mL^−1^) and intensity (a.u.) of the RAB3C expression model were examined by nanoparticle tracking analysis (NTA).
**Fig. S6.** Exosome concentration (particles·mL^−1^) and intensity (a.u.) of the RAB3C knockdown model were examined by nanoparticle tracking analysis (NTA).
**Fig. S7.** Relationships between RAB3C and interaction partners.
**Fig. S8.** RAB3C/dystrophin‐related immunoprecipitation analysis.
**Fig. S9.** Degradation rates between RAB3C and dystrophin.
**Fig. S10.** Various properties of dystrophin knockdown model validation.
**Fig. S11.** Exosome co‐culture validation.
**Fig. S12**. *In silico* analysis between RAB3C expression and chemotherapeutic drugs.
**Fig. S13.** Cell viability in SW480 RAB3C cells treated with different doses of predicted drugs.
**Fig. S14.** Cell viability under the treatment of regorafenib alone or combined various dosage of CB2 agonist AM1241 in SW480 vector cells.
**Fig. S15.** Multiplex immunofluorescence profiles in the RAB3C knockdown model.
**Fig. S16.** KRAS gene alterations and expression in colorectal cancer.
**Fig. S17.** The Kaplan–Meier plot show that patients with altered KRAS displayed poorer overall survival than those with unaltered group.
**Fig. S18.** The percentage of RAB3C expression levels in the wild‐type and mutant *APC and CTNNB1* groups.
**Fig. S19.** The correlation plot of IC50 of NVPBEZ235 and AZD6482 with RAB3C/dystrophin mRNA expression level in colon cancer cells.

**Video S1.** Extracellular vesicles flux of control group in CX‐1 cancer cells (10 s).

**Video S2.** Extracellular vesicles flux of RAB3C group in CX‐1 cancer cells (10 s).

**Video S3.** Extracellular vesicles flux of control group in DLD‐1 cancer cells (10 s).

**Video S4.** Extracellular vesicles flux of shRAB3C‐1 group in DLD‐1 cancer cells (10 s).

**Video S5.** Extracellular vesicles flux of shRAB3C‐2 group in DLD‐1 cancer cells (10 s).

## Data Availability

The datasets used and/or analyzed during the current study are available from the corresponding author on reasonable request.
