# Supplementary material for: Overexpression of synaptic vesicle protein Rab GTPase 3C promotes vesicular exocytosis and drug resistance in colorectal cancer cells
Source: Mol Oncol. 2023 Feb 14;17(3):422–44. doi: 10.1002/1878-0261.13378 (PMC9980308; doi:10.1002/1878-0261.13378)
Supplement: Supplementary file 1 — Table S1. Small compound candidates predicted by L1000 CDS2 in SW480 RAB3C cells. Table S2. Detailed resources and reagents used in this study. Table S3. List of common signatures in RAB3C‐based proteomics. Normalized to the vector control group and ordered by Log ratio. Table S4. Univariate and multivariate analyses for RAB3C/dystrophin expression in colorectal cancer. Fig. S1. Fraction collection after sucrose gradient separation and examination of exosome‐related markers. Fig. S2. Western blot analysis of RAB3C protein expression in various colon cancer cells and normal colon epithelial cells. Fig. S3. Western blots showed the RAB3C, total‐/phosphor‐Akt, RAB3B and RIMS1 in RAB3C knockdown models. Fig. S4. ROI region delineation and quantification of each group of confocal images. Fig. S5. Exosome concentration (particles·mL−1) and intensity (a.u.) of the RAB3C expression model were examined by nanoparticle tracking analysis (NTA). Fig. S6. Exosome concentration (particles·mL−1) and intensity (a.u.) of the RAB3C knockdown model were examined by nanoparticle tracking analysis (NTA). Fig. S7. Relationships between RAB3C and interaction partners. Fig. S8. RAB3C/dystrophin‐related immunoprecipitation analysis. Fig. S9. Degradation rates between RAB3C and dystrophin. Fig. S10. Various properties of dystrophin knockdown model validation. Fig. S11. Exosome co‐culture validation. Fig. S12. In silico analysis between RAB3C expression and chemotherapeutic drugs. Fig. S13. Cell viability in SW480 RAB3C cells treated with different doses of predicted drugs. Fig. S14. Cell viability under the treatment of regorafenib alone or combined various dosage of CB2 agonist AM1241 in SW480 vector cells. Fig. S15. Multiplex immunofluorescence profiles in the RAB3C knockdown model. Fig. S16. KRAS gene alterations and expression in colorectal cancer. Fig. S17. The Kaplan–Meier plot show that patients with altered KRAS displayed poorer overall survival than those with unaltered group. Fig. [file MOL2-17-422-s002.docx]

**Overexpression of synaptic vesicle protein Rab GTPase 3C promotes vesicular exocytosis and drug resistance in colorectal cancer cells**

Yu-Chan Chang^1*^, Chien-Hsiu Li^2*^, Ming-Hsien Chan^2^, Chih-Yeu Fang^3^, Zhi-Xuan Zhang^2^, Chi-Long Chen^4#^, Michael Hsiao^2,5#^

1. Department of Biomedical Imaging and Radiological Sciences, National Yang Ming Chiao Tung University, Taipei, Taiwan
2. Genomics Research Center, Academia Sinica, Taipei, Taiwan
3. National Institute of Infectious Diseases and Vaccinology, National Health Research Institutes, Miaoli, Taiwan
4. Department of Pathology, Taipei Medical University Hospital and College of Medicine, Taipei Medical University, Taipei, Taiwan
5. Department of Biochemistry, College of Medicine; Kaohsiung Medical University; Kaohsiung, Taiwan

*the first two authors contribute equally to this work

^#^To whom correspondence should be addressed:

Dr. Michael Hsiao, Genomics Research Center, Academia Sinica, 128 Academia Rd., Sec. 2, Nankang-Dist., Taipei, Taiwan. Tel: +886-2-2787-1243, Fax: +886-2-2789-9931, E-mail: [mhsiao@gate.sinica.edu.tw](mailto:mhsiao@gate.sinica.edu.tw)

**Supplementary Table 1.** Small compound candidates predicted by L1000 CDS^2^ in SW480 RAB3C cells.

| **Overlap** | **Perturbation** | **Cell-line** | **Dose** | **Time** | **Note** |
| --- | --- | --- | --- | --- | --- |
| 0.2016 | IMD 0354 | MCF7 | 10.0um | 24.0h | IKK2 inhibitor |
| 0.186 | phorbol-12-myristate-13-acetate (PMA) | MCF7 | 10.0um | 24.0h | PKC activator |
| 0.186 | Ingenol 3 |  |  |  | inhibit T cell apoptosis |
| 0.186 | phorbol-12-myristate-13-acetate (PMA) | SW948 | 10.0um | 6.0h | PKC activator |
| 0.1783 | phorbol-12-myristate-13-acetate (PMA) | A549 | 10.0um | 6.0h | PKC activator |
| 0.1783 | BRD-K94325918 | MCF7 | 10.0um | 24.0h | anti-proliferative agent and induces apoptosis |
| 0.1783 | BIBR1532 | SW620 | 80.0um | 6.0h | telomerase inhibitor |
| 0.1628 | curcubitacin I | HT29 | 10.0um | 24.0h | STAT3/JAK inhibitor |
| 0.1628 | Rottlerin | MCF7 | 9.68um | 24.0h | PKCdelta inhibitor |
| 0.1628 | METHYLENE BLUE | MCF7 | 10.0um | 24.0h |  |
| 0.1628 | BRD-K20755323 | MCF7 | 40.0um | 24.0h |  |
| 0.1628 | BRD-K92317137 | MCF7 | 10.0um | 24.0h |  |
| 0.1628 | BRD-K91145395 | HCC515 | 10.0um | 6.0h | inhibited in vitro HIV-1 replication |
| 0.1628 | BRD-K91145395 | MCF7 | 10.0um | 6.0h | inhibited in vitro HIV-1 replication |
| 0.155 | phorbol-12-myristate-13-acetate (PMA) | AGS | 10.0um | 6.0h | PKC activator |
| 0.155 | Ingenol 3 |  |  |  | inhibit T cell apoptosis |
| 0.155 | phorbol-12-myristate-13-acetate (PMA) | HCC515 | 10.0um | 6.0h | PKC activator |
| 0.155 | Ingenol 3 |  |  |  | inhibit T cell apoptosis |
| 0.155 | Emetine Dihydrochloride Hydrate (74) | HT29 | 0.63um | 24.0h | Emetine inhibits protein synthesis in eukaryotic cells by binding to the 40S ribosomal subunit and inhibiting translocation |
| 0.155 | GW405833 hydrochloride | HT29 | 40.0um | 24.0h | CB2 agonist |
| 0.155 | Salermide | HT29 | 120.0um | 6.0h | SIRT1/SIRT2 inhibitor |
| 0.155 | phorbol-12-myristate-13-acetate (PMA) | SW620 | 10.0um | 6.0h | PKC activator |
| 0.155 | Ro 28-1675 ? | SW620 | 160.0um | 6.0h | Glucokinase activator |
| 0.155 | CGK-733 | HEPG2 | 10um | 24h | ATM/ATR inhibitor |
| 0.1473 | BRD-K28907958 | HA1E | 11.1um | 24.0h |  |
| 0.1473 | Salermide | HA1E | 120.0um | 6.0h | sirtuin inhibitor |
| 0.1473 | METHYLENE BLUE | HT29 | 10.0um | 24.0h |  |
| 0.1473 | Ro 28-1675 ? | HT29 | 160.0um | 24.0h | Glucokinase activator |
| 0.1473 | BRD-K66792149 | HT29 | 11.1um | 24.0h | inhibited NF-KB activity |
| 0.1473 | NICLOSAMIDE | MCF7 | 10.0um | 24.0h | STAT3 inhibitor |
| 0.1473 | B675700.cdx | MCF7 | 0.04um | 24.0h |  |
| 0.1473 | Ingenol 3 |  |  |  | inhibit T cell apoptosis |
| 0.1473 | phorbol-12-myristate-13-acetate (PMA) | RMUGS | 10.0um | 6.0h | PKC activator |
| 0.1473 | S1130 | HT29 | 10.0um | 6.0h | suppress survivin transactivation |
| 0.1395 | RHODOMYRTOXIN B | A375 | 10.0um | 24.0h | GPD2 inhibitor |
| 0.1395 | BRD-K19295594 | A375 | 11.1um | 24.0h | antispermatogenic |
| 0.1395 | phorbol-12-myristate-13-acetate (PMA) | CL34 | 10.0um | 6.0h | PKC activator |
| 0.1395 | phorbol-12-myristate-13-acetate (PMA) | HA1E | 10.0um | 6.0h | PKC activator |
| 0.1395 | "Ingenol 3 |  |  |  | inhibit T cell apoptosis |
| 0.1395 | BRD-K43620258 | HT29 | 80.0um | 24.0h |  |
| 0.1395 | curcubitacin I | HT29 | 10.0um | 6.0h | STAT3/JAK inhibitor |
| 0.1395 | Emetine Dihydrochloride Hydrate (74) | HT29 | 0.63um | 6.0h | Emetine inhibits protein synthesis in eukaryotic cells by binding to the 40S ribosomal subunit and inhibiting translocation |
| 0.1395 | BRD-K17140735 | MCF7 | 11.1um | 24.0h | PAR1 inhibitor |
| 0.1395 | TW 37 | MCF7 | 10.0um | 24.0h |  |
| 0.1395 | Ro 28-1675 ? | PC3 | 160.0um | 24.0h | Glucokinase activator |
| 0.1395 | BRD-K64642496 | PC3 | 40.0um | 24.0h |  |
| 0.1395 | BRD-K68143200 | SW620 | 10.0um | 6.0h |  |
| 0.1395 | CHR 2797 | SW620 | 80.0um | 6.0h | antiproliferative aminopeptidase inhibitor |
| 0.1395 | BRD-A34205397 | MCF7 | 10.0um | 24.0h | inhibiti of platelet aggregation |
| 0.1395 | PLX-4720 | HEPG2 | 10um | 24h | BRAF inhibitor |

| REAGENT or RESOURCE | sOURCE | IDENTIFIER |
| --- | --- | --- |
| **Antibodies** |  |  |
| Rabbit polyclonal anti-RAB3C (western blot 1:5000) | GeneTex | GTX108016 |
| Rabbit polyclonal anti-RAB3C (IHC 1:200) | Proteintech | Cat # 15029-1-AP |
| Rabbit polyclonal anti-Dystrophin (western blot 1:1000) | Atlas antibodies | Cat # HPA023885 |
| Rabbit anti-PIK3CA E545K (western blot1:1000 /IF 1:100) | NewEast | Cat# No. 21146 |
| Rabbit anti-PIK3CA H1047R (western blot1:1000 /IF 1:100) | NewEast | Cat# No. 21145 |
| Rabbit monoclonal anti-phospho-Akt (S473) (western blot1:1000 /IF 1:200) | Cell Sgiangling | Cat # 4060 |
| Rabbit anti-RAB3B (western blot1:1000 /IF 1:200) | GeneTex | GTX108610 |
| Rabbit anti-EXO1 (western blot1:1000 /IF 1:200) | GeneTex | GTX9891 |
| Mouse anti-IgG (western blot1:1000 ) | Cell Signaling | Cat# No. 7076 |
| Mouse monoclonal anti-α-Tubulin (western blot 1:5000) | Sigma-Aldrich | Cat# No. T5168 |
| **Critical Commercial Assays** |  |  |
| Alamar Blue reagent | ThermoFisher | Cat# No.DAL1025 |
| **Chemicals, Peptides, and Recombinant Proteins** |  |  |
| CB2 agonist | Cayman Chemical | AM1241 |
| CB2 inverse agonist | Abcam | BML-190 |
| Regorafenib | Bayer | Stivarga^Ⓡ^ |
| **Biological Samples** |  |  |
| Human tissue samples | Taipei Medical University | Dr. Chi-Long Chen |
| **Experimental Model** |  |  |
| Microarray chip | Affymetrix | Human Genome U133 plus 2.0 |
| **Software and Algorithms** |  |  |
| SPSS Statistics | IBM | Version 20.0.0 |
| GraphPad Prism | GraphPad Software | Version 5.0.1 |
| Adobe Photoshop CS2 | Adobe | Version 9.0 |
| Connectivity-Map (L1000CDS2) | Ma’ayan Laboratory |  |
| ImageScope | Aperio | Version 12.2.2.5015 |

**Supplementary Table 2.** Detailed resources and reagents used in this study.

**Supplementary Table 3.** List of common signatures in RAB3C-based proteomics. Nomrlized to the vector control group and ordered by Log ratio.

| **Up** | | |
| --- | --- | --- |
| DMD/Dystrophin (6.64) | RAB3C (6.48) | PDHB (6.06) |
| PLEC (5.76) | PUS1 (5.60) | PTPLAD1/HACD3 (5.56) |
| ACT1/TRAF3IP2 (5.34) |  |  |
| **Down** | | |
| TSPAN8 (-6.83) | RPL27A (-6.66) | ACTBL2 (-6.65) |
| RPL35 (-6.60) | SUMO3 (-6.58) | RPL17 (-6.53) |
| PFN2 (-6.45) | DSTN (-6.23) | ARF5 (-6.23) |
| ARF5 (-6.23) | EWSR1 (-6.21) | SPINT2 (-6.16) |
| BRI3BP (-6.14) | RPL9B (-6.13) | RAB21 (-6.03) |
| VPS29 (-5.98) | RPL21 (-5.96) | CSTB (-5.94) |
| ACTR1B (-5.90) | PPP1CC (-5.88) | NOP56 (-5.88) |
| TMEM109 (-5.86) | AIMP2 (-5.84) | ADSS (-5.84) |
| ABHD10 (-5.82) | NIT2 (-5.81) | GNG12 (-5.80) |
| ATIC (-5.80) | ALDH9A (-5.77) | BIP1 (-5.75) |
| DNPEP (-5.73) | VAMP2 (-5.72) | TRA2A (-5.69) |
| NIPSNAP1 (-5.69) | TATDN1 (-5.69) | TWF1 (-5.68) |
| SCAMP2 (-5.65) | HNRNPA0 (-5.63) | MRPS22 (-5.58) |
| PDL1M1 (-5.58) | NOLC1 (-5.58) | PABPN1 (-5.57) |
| TMED10 (-5.56) | FAF1 (-5.55) | PKP2 (-5.48) |
| NUP37 (-5.43) | SNRPB (-5.41) | PSMC3 (-5.39) |
| GTF2F1 (-5.35) | NCKAP1 (-5.34) | MRPL37 (-5.33) |
| SHMT1 (-5.28) | ASPH (-5.26) | XPO7 (-5.17) |
| MCM7 (-5.16) |  |  |

**Supplementary Table 4.** Univariate and multivariate analyses for RAB3C/dystrophin expression in colorectal cancer.

| Cox univariate analysis (OS) | | |  |  |  |
| --- | --- | --- | --- | --- | --- |
| Variables | | Comparison | HR (95% CI) | | *P*-value |
| T |  | T3-T4 vs. T1-T2 | 3.241 (1.422-7.384) | | 0.005 |
| N |  | N1-N3 vs. N0 | 1.364 (0.936-1.989) | | 0.106 |
| M |  | M1 vs. M0 | 6.383 (4.117-9.896) | | 1.2e-16 |
| RAB3C+Dystrophin |  | High vs. Low | 1.463 (1.124-1.905) | | 0.005 |
| Cox multivariate analysis (OS) | | |  |  |  |
| Variables | | Comparison | HR (95% CI) | | *P*-value |
| T |  | T3-T4 vs. T1-T2 | 2.428 (1.048-5.626) | | 0.039 |
| N |  | N1-N3 vs. N0 | 1.227 (0.835-1.802) | | 0.298 |
| M |  | M1 vs. M0 | 5.468 (3.512-8.514) | | 5.5e-14 |
| RAB3C+Dystrophin |  | High vs. Low | 1.407 (1.074-1.843) | | 0.013 |
| Cox univariate analysis (DFS) | | |  |  |  |
| Variables | | Comparison | HR (95% CI) | | *P*-value |
| T |  | T3-T4 vs. T1-T2 | 4.385 (1.192-13.938) | | 0.012 |
| N |  | N1-N3 vs. N0 | 1.814 (1.478-2.931) | | 0.015 |
| M |  | M1 vs. M0 | 11.231 (6.843-18.432) | | 1.1e-21 |
| RAB3C+Dystrophin |  | High vs. Low | 1.419 (1.025-1.966) | | 0.035 |
| Cox multivariate analysis (DFS) | | |  |  |  |
| Variables | | Comparison | HR (95% CI) | | *P*-value |
| T |  | T3-T4 vs. T1-T2 | 2.464 (0.757-8.013) | | 0.134 |
| N |  | N1-N3 vs. N0 | 1.574 (0.968-2.559) | | 0.068 |
| M |  | M1 vs. M0 | 9.616 (5.789-15.973) | | 2.3e-18 |
| RAB3C+Dystrophin |  | High vs. Low | 1.260 (0.892-1.780) | | 0.190 |

**Supplementary Video 1.** Extracellular vesicles flux of control group in CX-1 cancer cells (10s).

**Supplementary Video 2.** Extracellular vesicles flux of RAB3C group in CX-1 cancer cells (10s).

**Supplementary Video 3.** Extracellular vesicles flux of control group in DLD-1 cancer cells (10s).

**Supplementary Video 4.** Extracellular vesicles flux of shRAB3C-1 group in DLD-1 cancer cells (10s).

**Supplementary Video 5.** Extracellular vesicles flux of shRAB3C-2 group in DLD-1 cancer cells (10s).





**Supplementary Figure S1.** Fraction collection after sucrose gradient separation and examination of exosome-related markers.

**
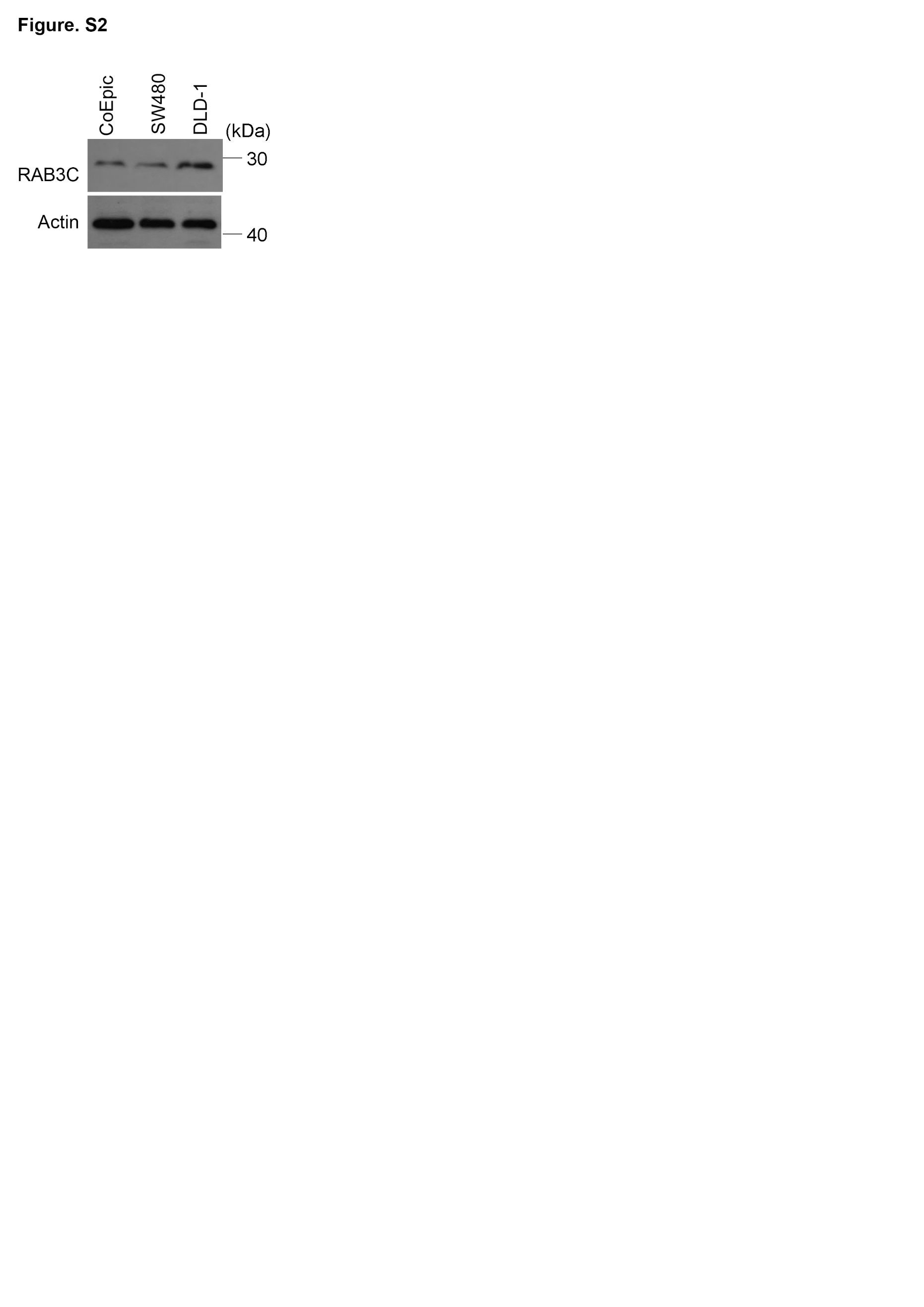
**

**Supplementary Figure S2.** Western blot analysis of RAB3C protein expression in various colon cancer cells and normal colon epithelial cells.





**Supplementary Figure S3.** Western blots showed the RAB3C, total-/phosphor-Akt, RAB3B and RIMS1 in RAB3C knockdown models.


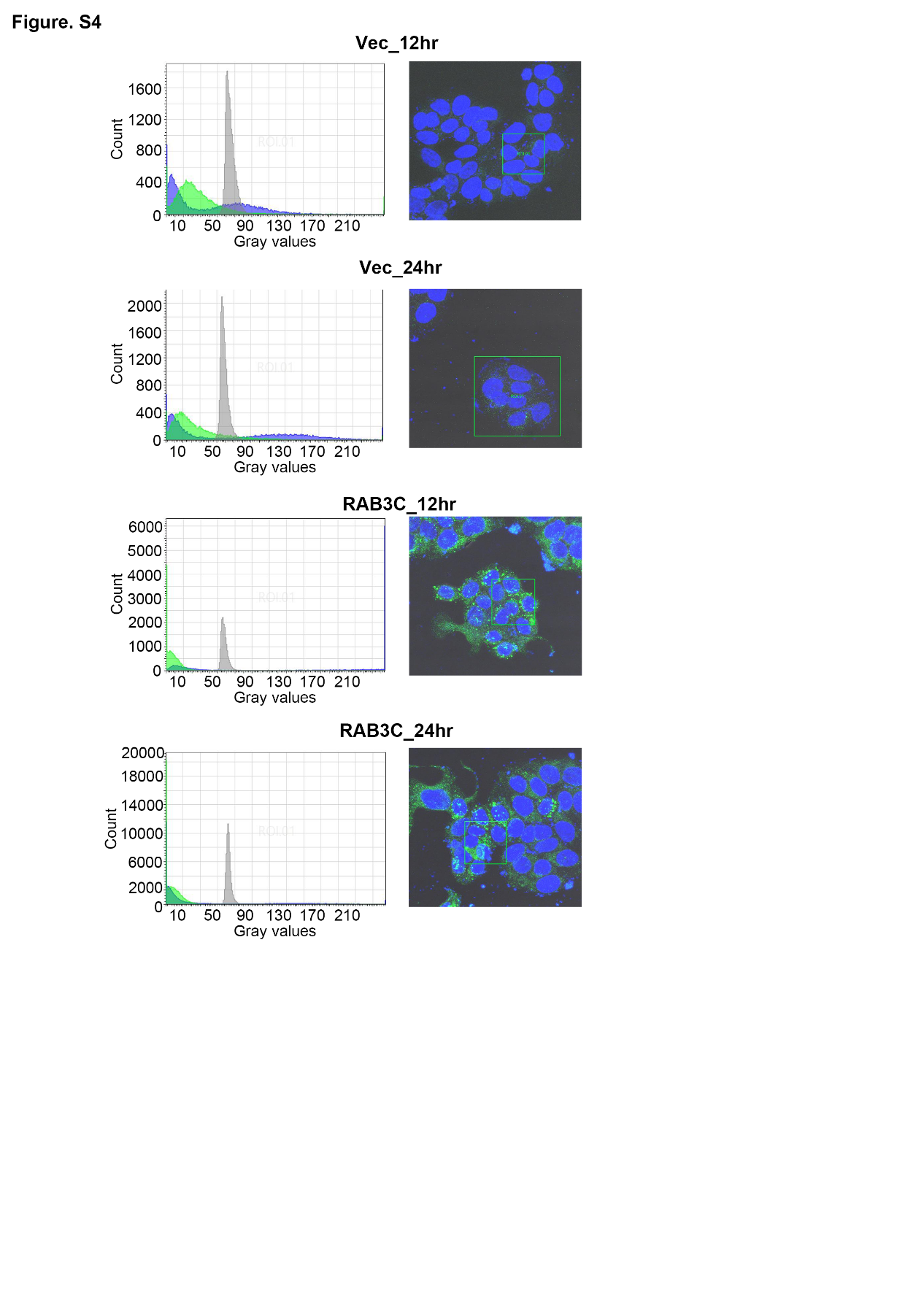


**Supplementary Figure S4.** ROI region delineation and quantification of each group of confocal images.

**
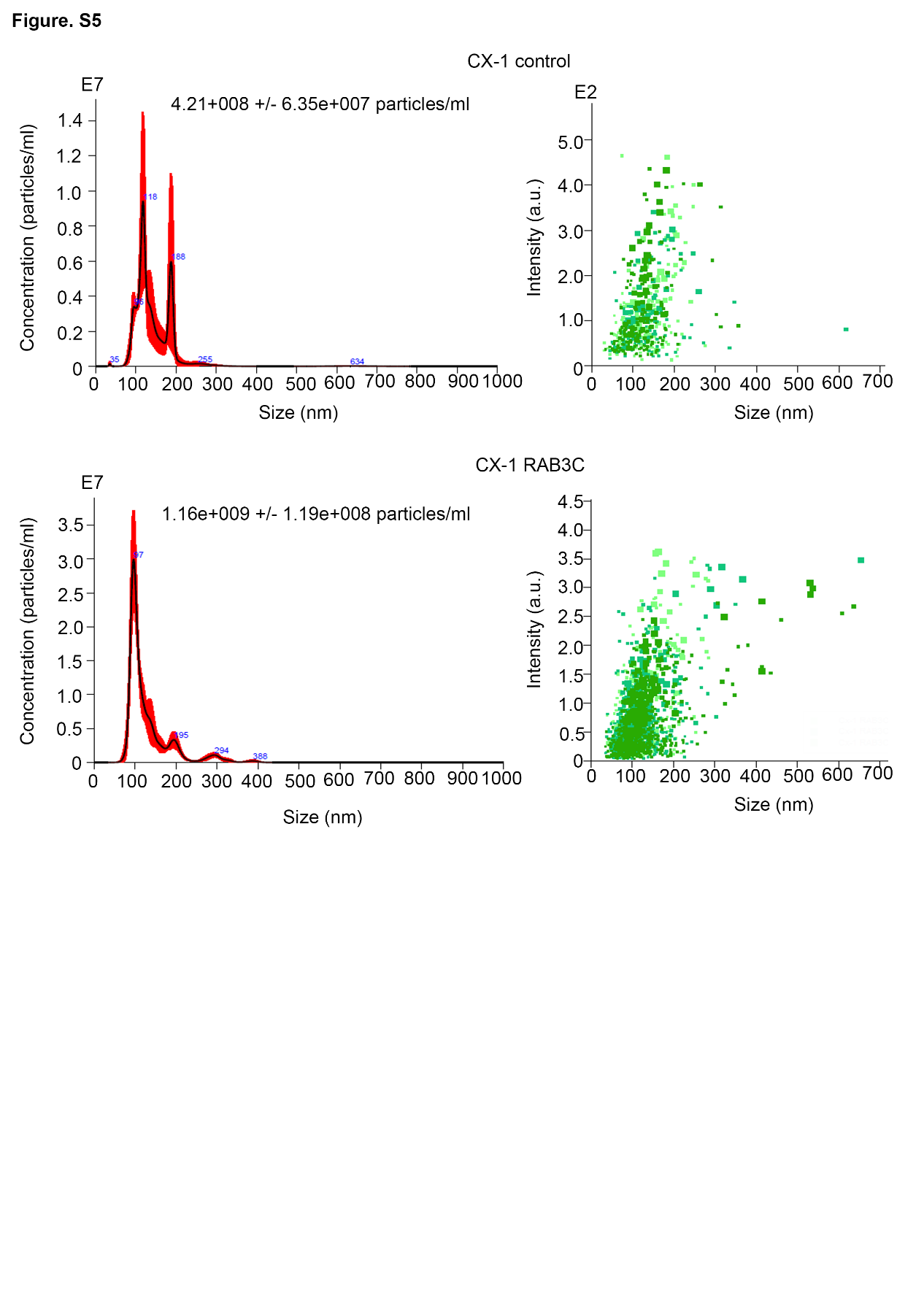
**

**Supplementary Figure S5.** Exosome concentration (particles/ml) and intensity (a.u.) of the RAB3C expression model were examined by nanoparticle tracking analysis (NTA).


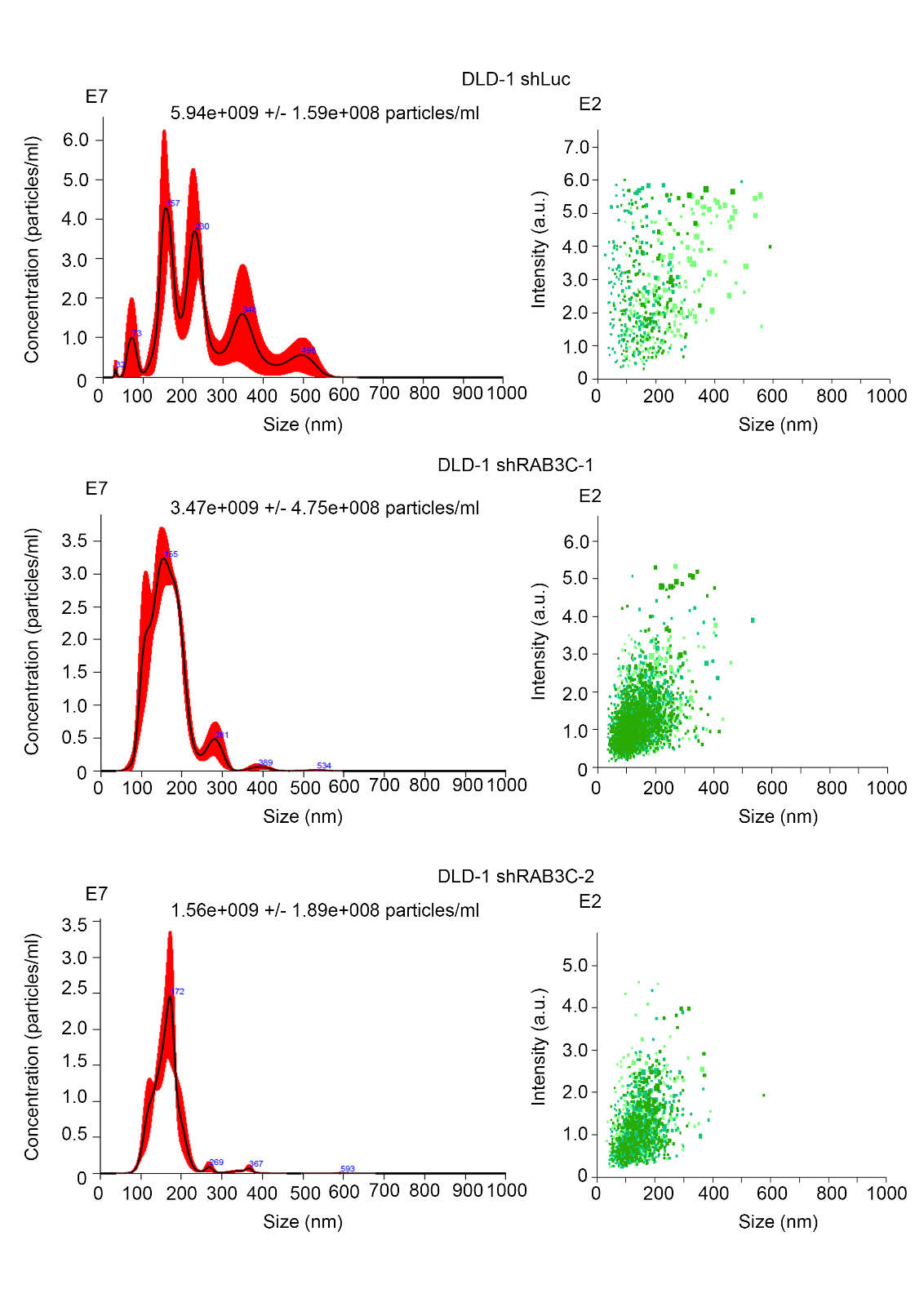
 **Supplementary Figure S6.** Exosome concentration (particles/ml) and intensity (a.u.) of the RAB3C knockdown model were examined by nanoparticle tracking analysis (NTA).


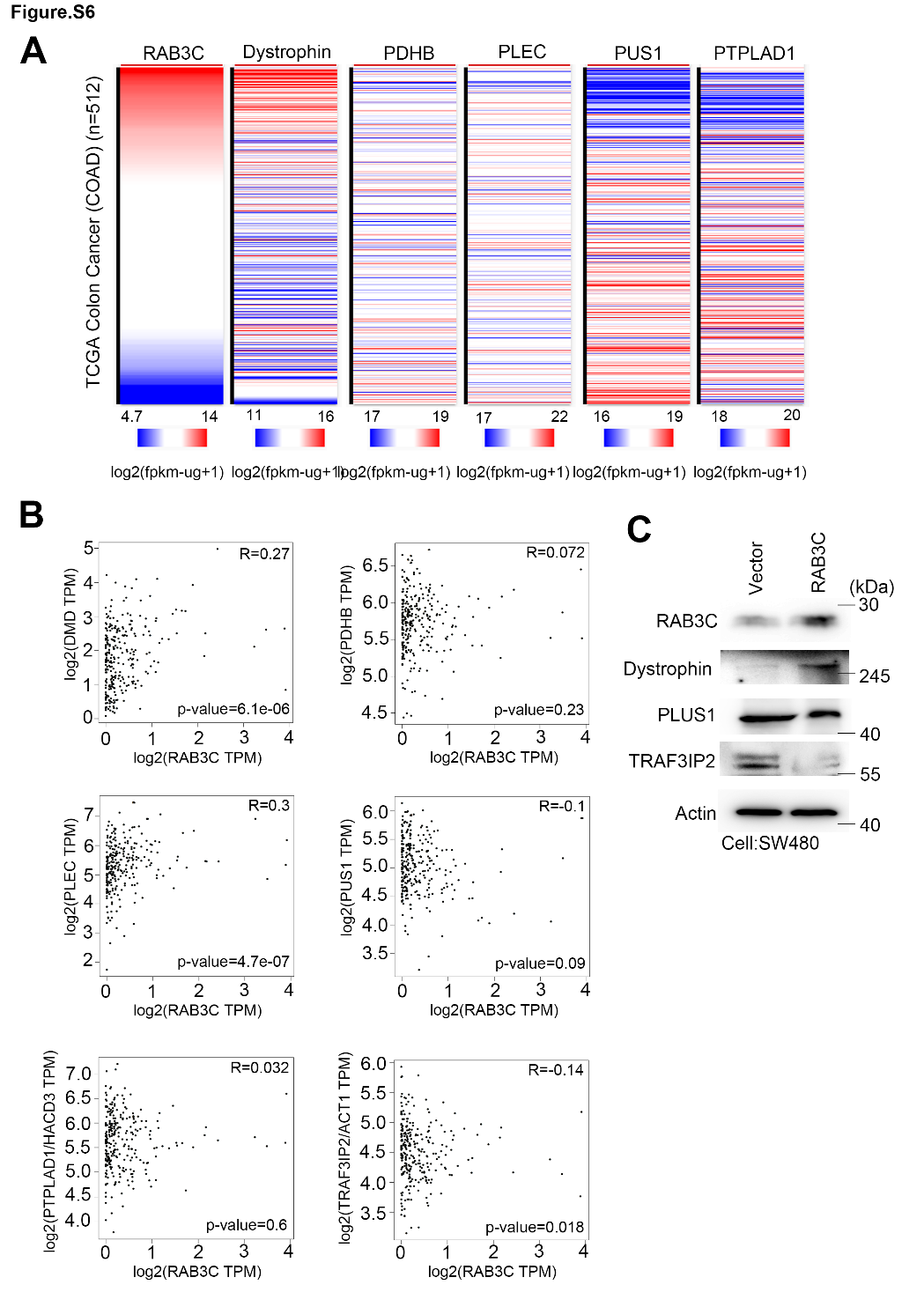


**Supplementary Figure S7.** Relationships between RAB3C and interaction partners. (A) Heatmap showing the expression level of RAB3C and other molecules in the TCGA_colorectal cancer cohort (n=512). (B) The correlation plot showed that the expression levels in the TCGA_colorectal cancer cohort. (C) Western blot analysis of the expression of various candidate proteins in the RAB3C overexpression model.


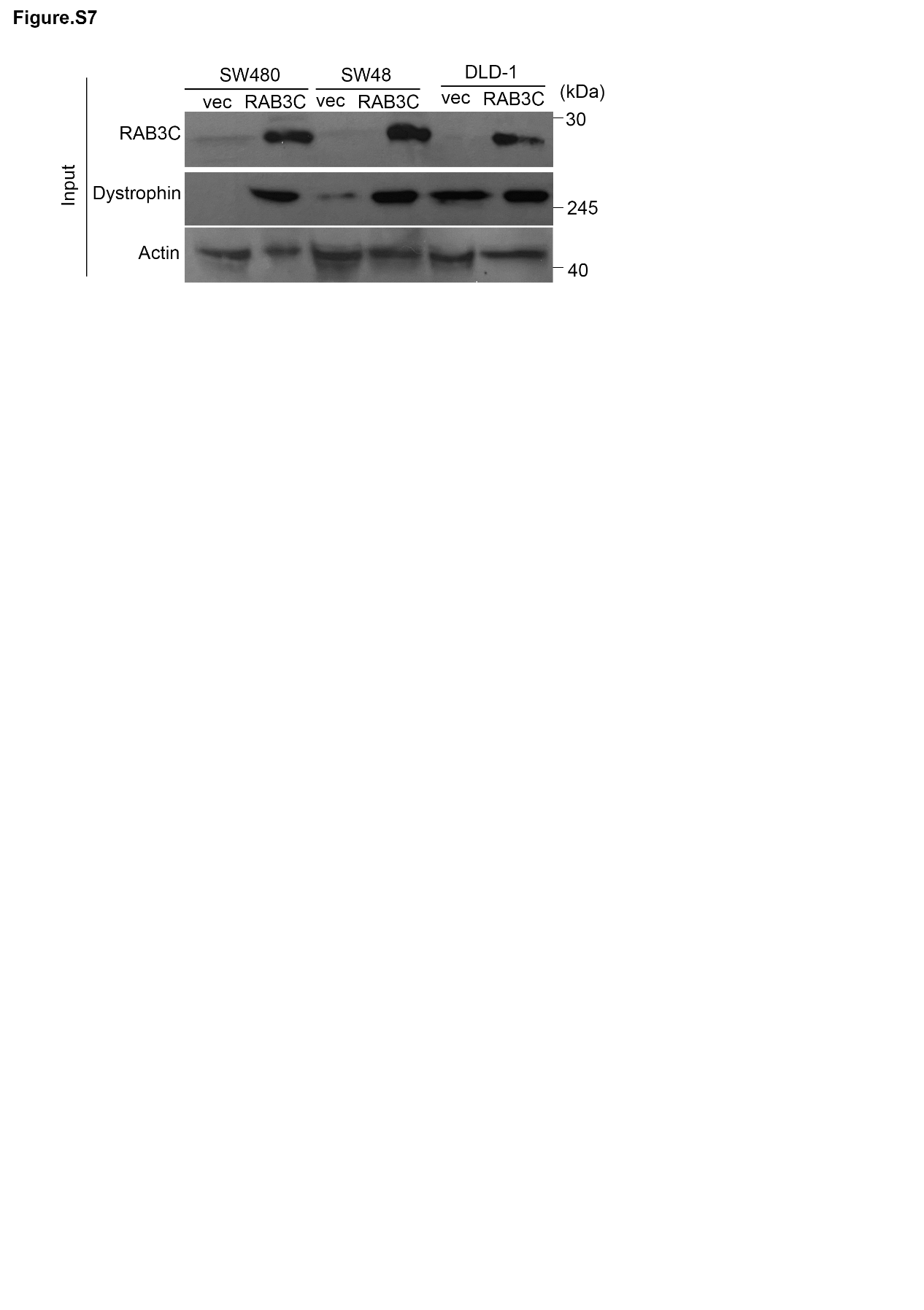


**Supplementary Figure S8.** RAB3C/dystrophin-related immunoprecipitation analysis. Results are performed on whole cell lysates from SW480, SW48 and DLD-1 RAB3C overexpression models.

**
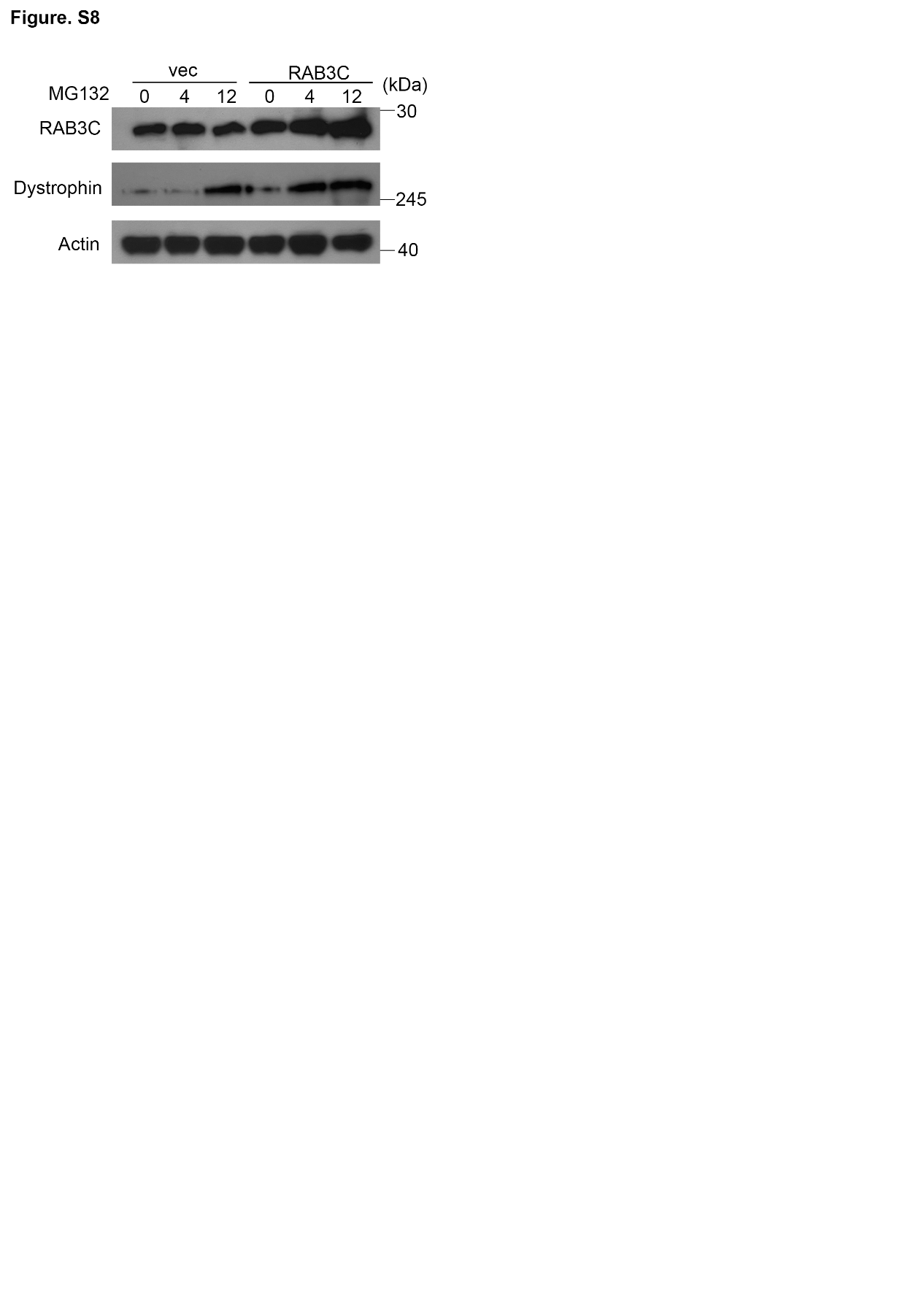
**

**Supplementary Figure S9.** Degradation rates between RAB3C and dystrophin. The expression of RAB3C and dystrophin proteins in the RAB3C overexpression model with or without treatment with MG-132 was analyzed by western blot. The concentration of MG-132 is 1.5μM, time: 0. 4 and 12 hours.

**
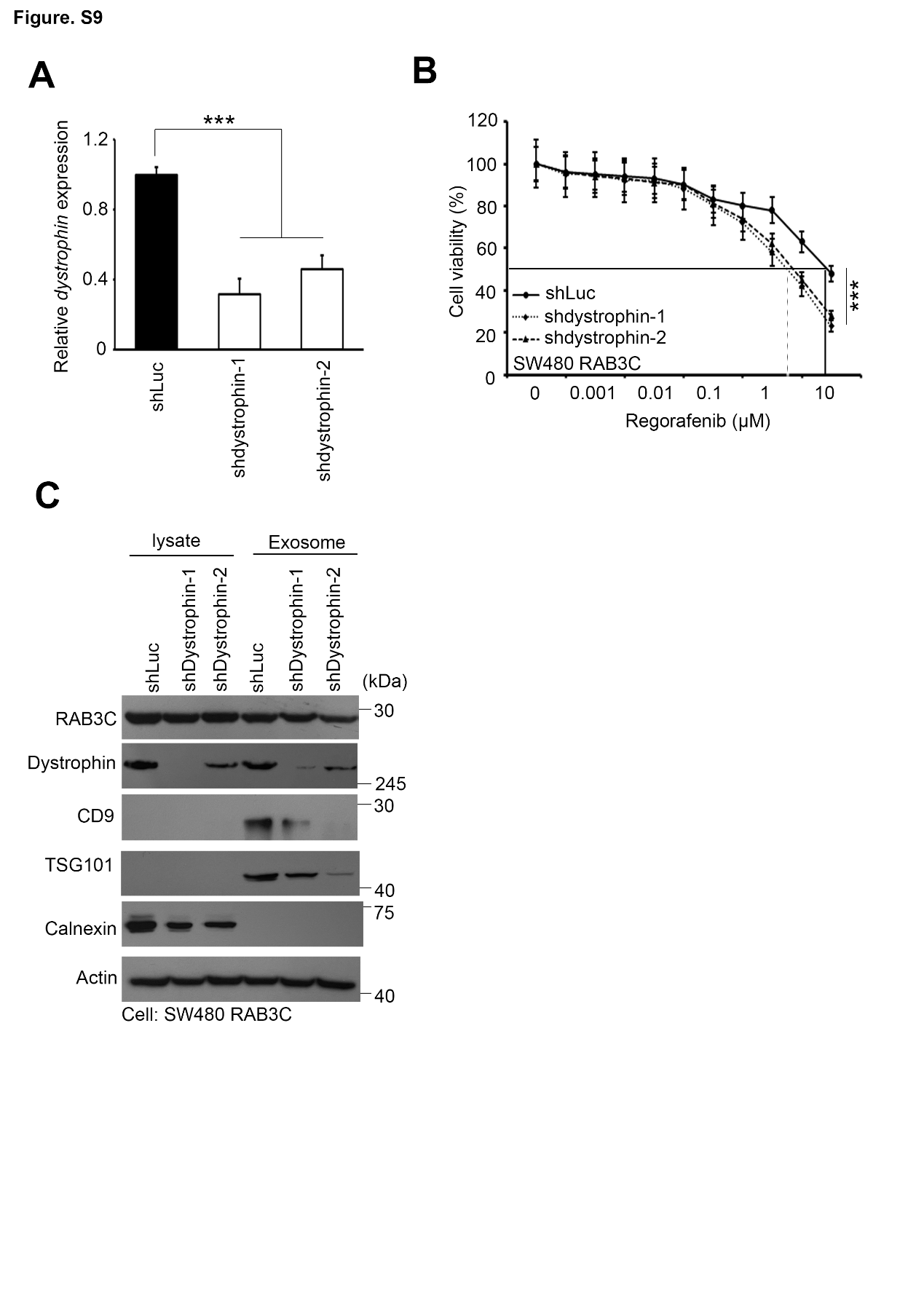
**

**Supplementary Figure S10.** Various properties of dystrophin knockdown model validation. **(A)** qRT-PCR analysis of *dystrophin* expression in the RAB3C expression model with or without dystrophin shRNAs. **(B)** Cell viability of regorafenib treatment in the RAB3C expression model with or without dystrophin shRNAs. **(C)** Western blot analysis of the expression of RAB3C, dystrophin, CD9, TSG101, and calnexin of whole cells and exosomes isolated in the RAB3C expression model with or without dystrophin knockdown.


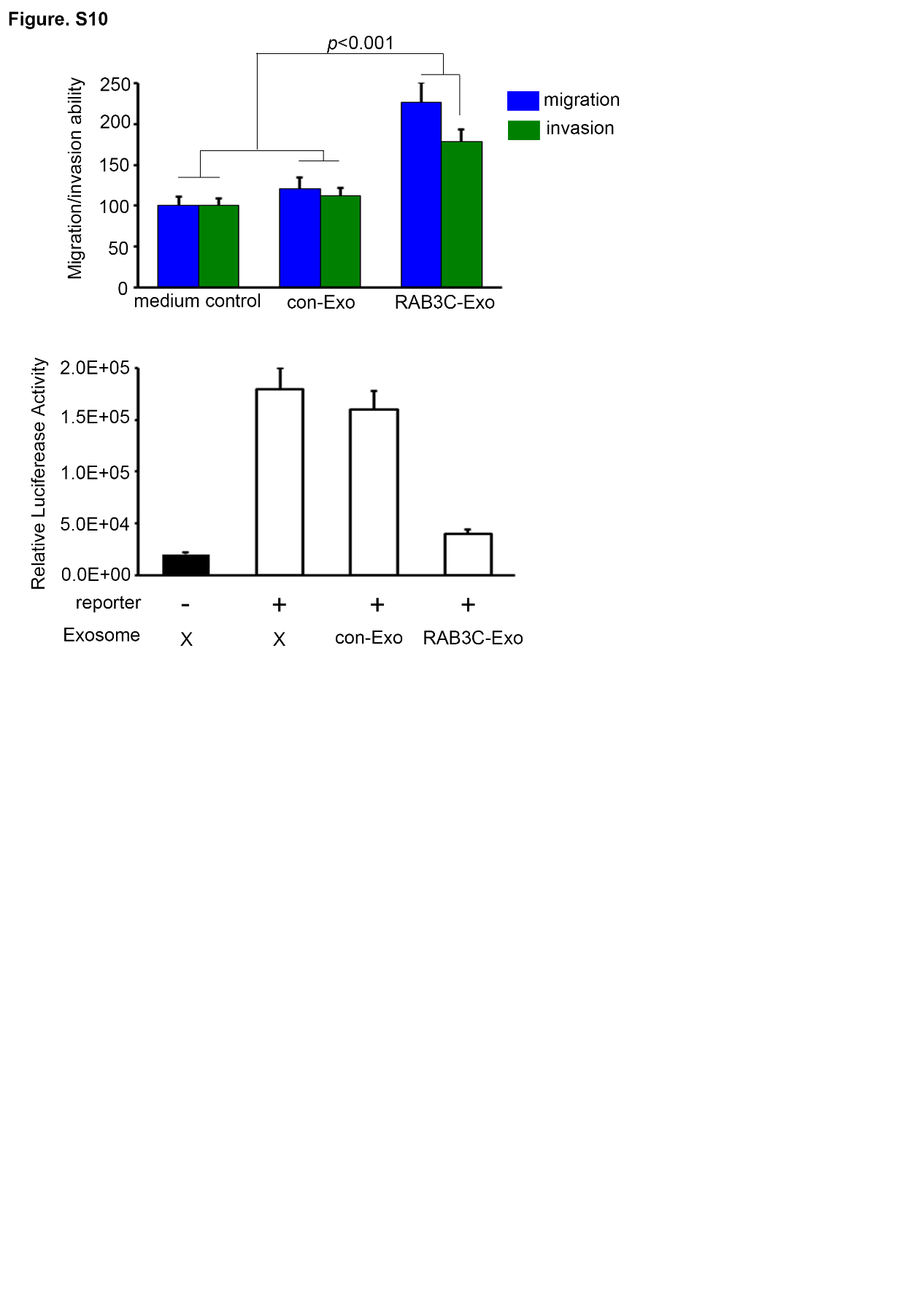


**Supplementary Figure S11.** Exosome co-culture validation. (upper) Detecting the migration and invasion ability of co-cultures with or without exosomes in SW480 cells. (lower) The E-cadherin reporter activity measured in co-cultures with or without exosomes in SW480 cells.


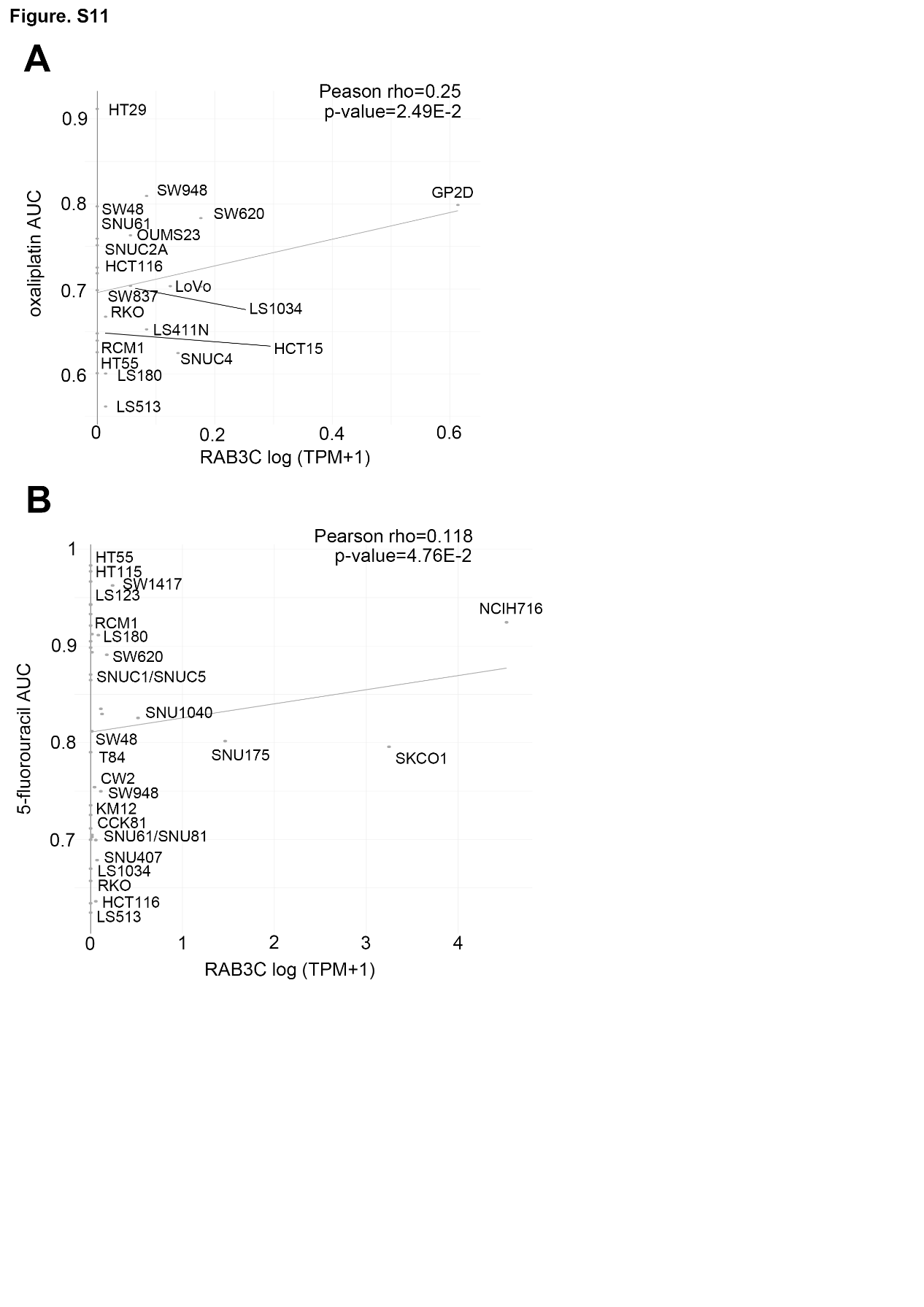


**Supplementary Figure S12**. *In silico* analysis between RAB3C expression and chemotherapeutic drugs. (A) The correlation graph shows the relationship between the expression level (Expression 21Q4 public) and the IC50 of oxaliplatin (BRD:BRD-K78960041-001-05-7) from the DepMap portal website. Pearson rho=0.25, *p*-value=0.0249. (B) The correlation plot shows the relationship between the RAB3C expression level of RAB3C (Expression 22Q2 public) and the IC50 of 5-fluorouracil (GDSC1:179) from the DepMap portal website. Pearson rho=0.12, *p*-value=0.0476.

**
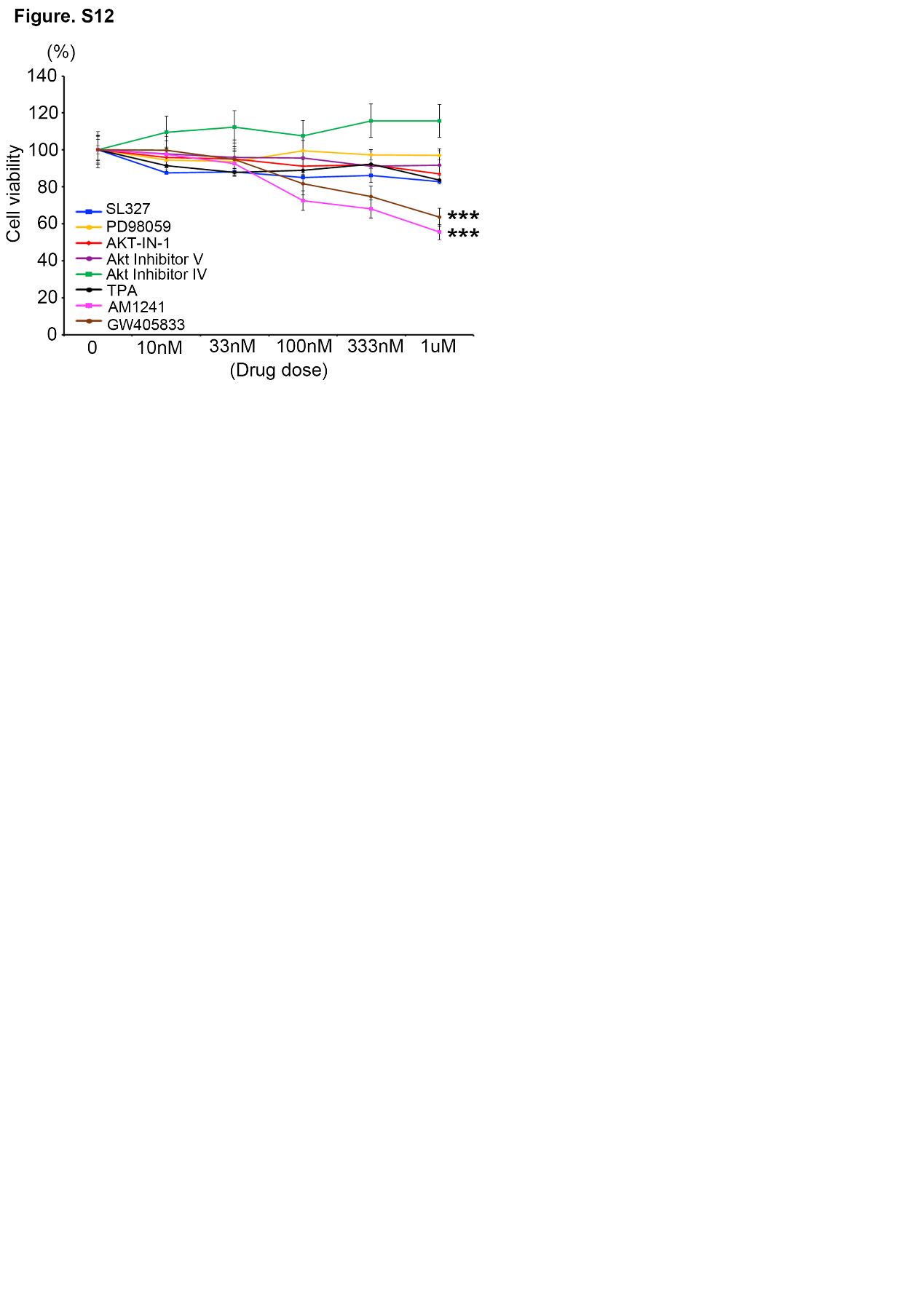
**

**Supplementary Figure S13.** Cell viability in SW480 RAB3C cells treated with different doses of predicted drugs (10nM-1μM) in Figure 3D.


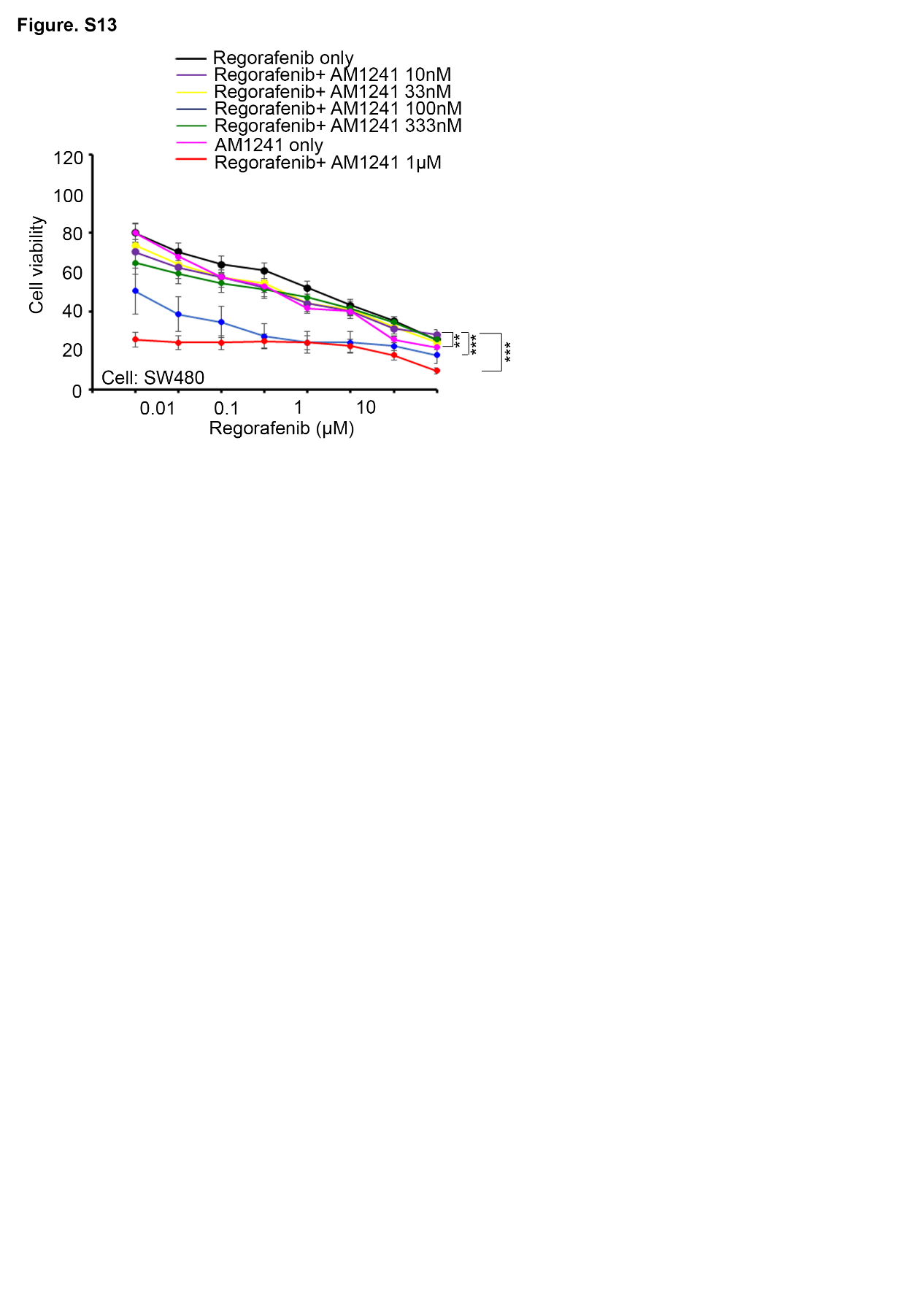


**Supplementary Figure S14.** Cell viability under the treatment of regorafenib alone or combined various dosage of CB2 agonist AM1241 (10nM-1μM) in SW480 vector cells.


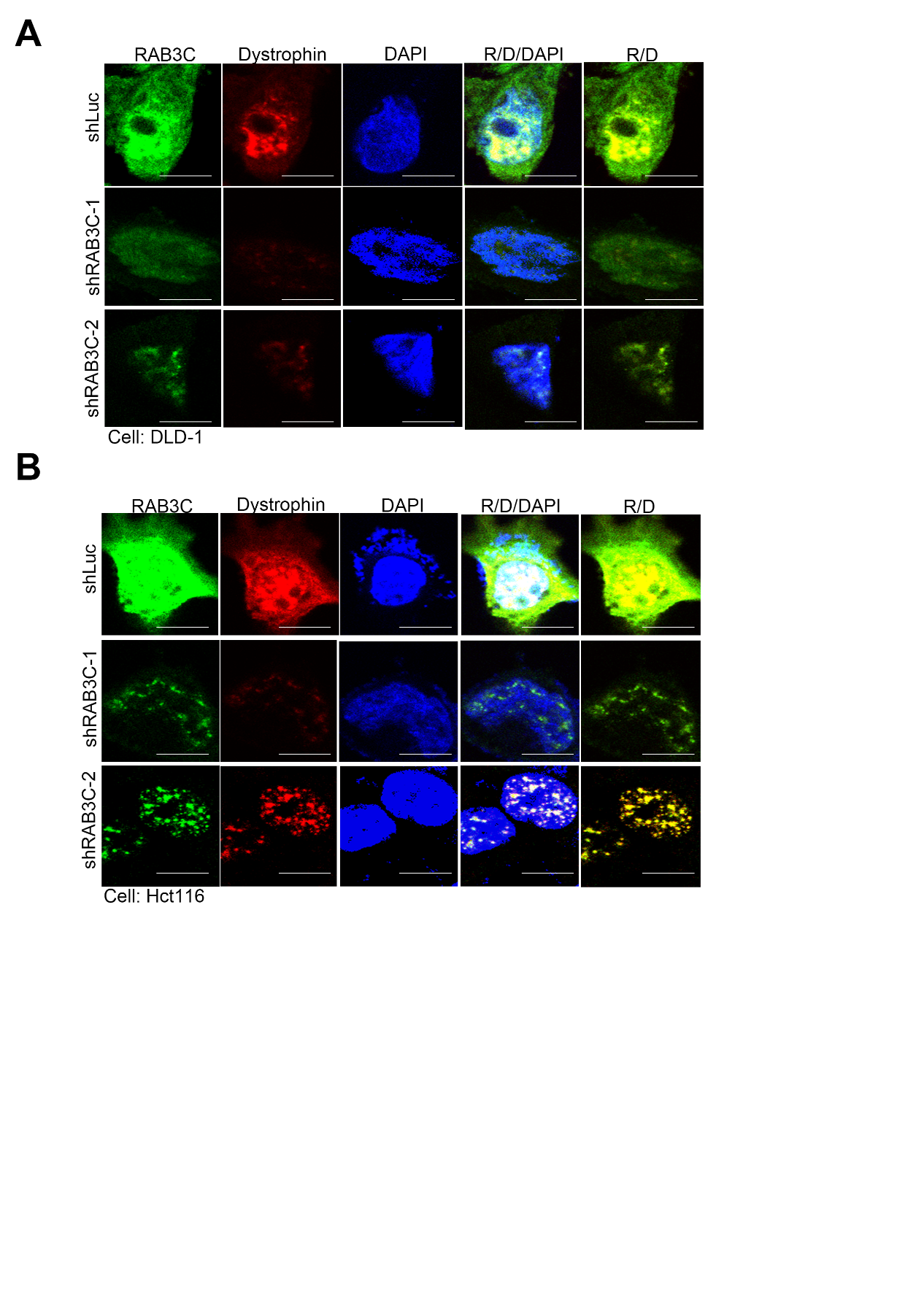


**Supplementary Figure S15.** Multiplex immunofluorescence profiles in the RAB3C knockdown model. (A) Representative fluorescence images of dystrophin/RAB3C protein staining results in the DLD-1 RAB3C knockdown model. (B) Representative fluorescence images of dystrophin/RAB3C protein staining results in the Hct116 RAB3C knockdown model. Scale bar: 10μM.


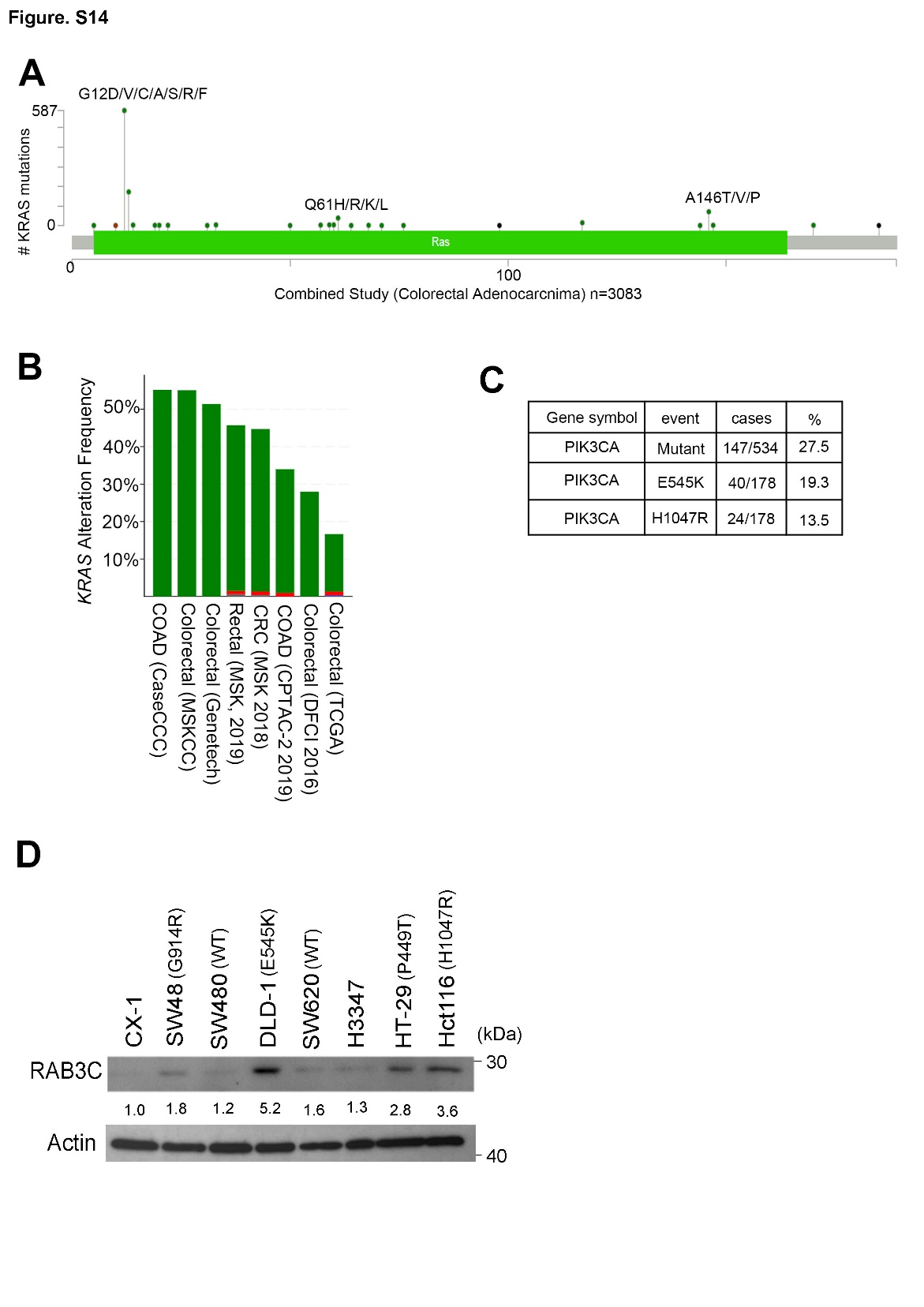


**Supplementary Figure S16.** KRAS gene alterations and expression in colorectal cancer. (A) The plot shows the protein domains of *KRAS* and its mutation hot-spots in colorectal adenocarcinoma patients from the TCGA clinical cohort (n = 3083) from GEPIA (B) The bar graph represents the frequencies of *KRAS* alteration displayed in multiple clinical cohorts. Green: gene mutation. Red: amplification.(C) Collection and summary of all of the genetic events of *PIK3CA*, specifically the E545K or H1047R mutants of *PIK3CA* in TCGA cohort. (D) Western blot analysis of RAB3C protein expression and its corresponding *PIK3CA* gene alteration events in colorectal cancer cells.


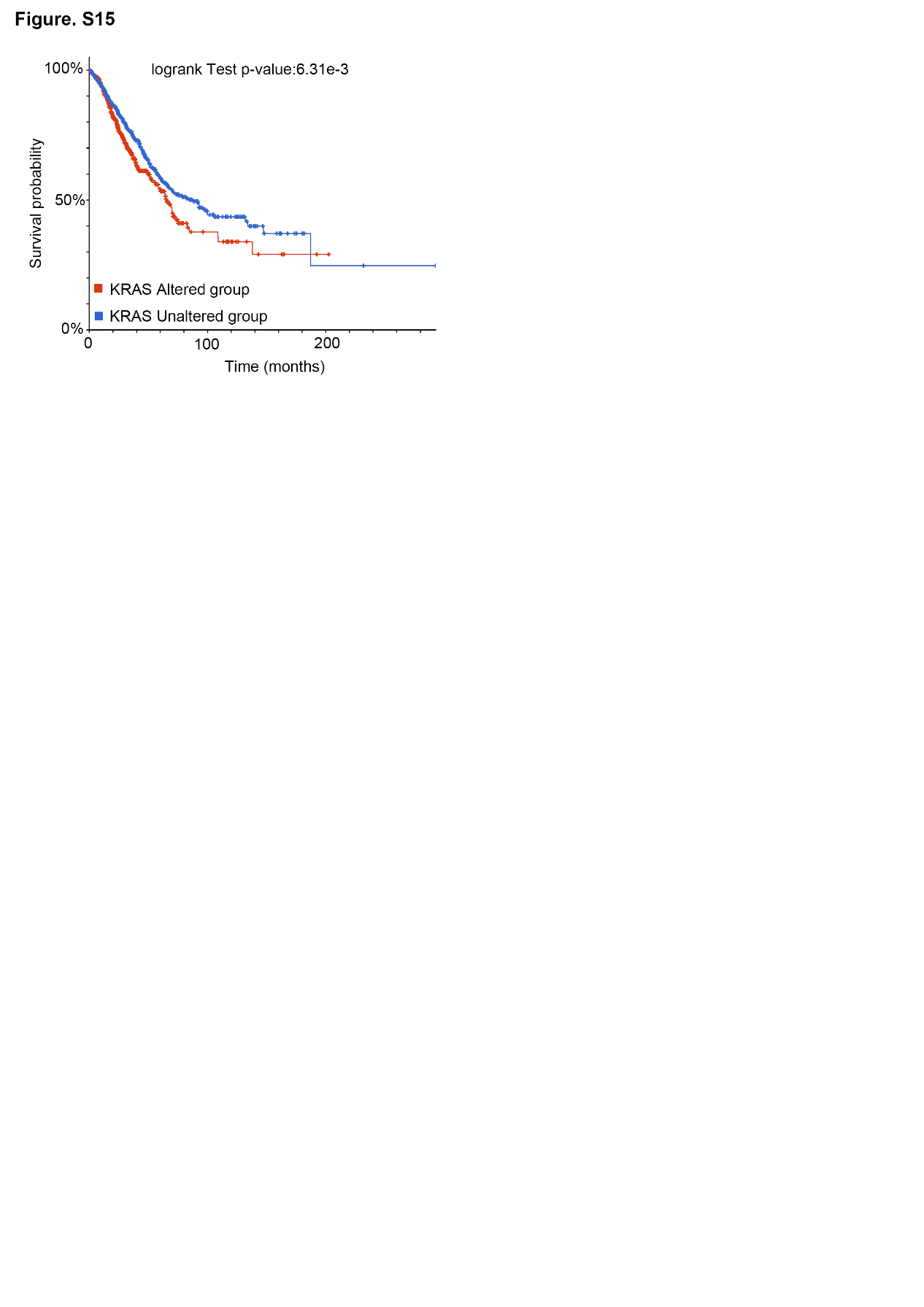


**Supplementary Figure S17.** The Kaplan-Meier plot show that patients with altered KRAS displayed poorer overall survival than those with unaltered group (*p*=6.3e-3).

**
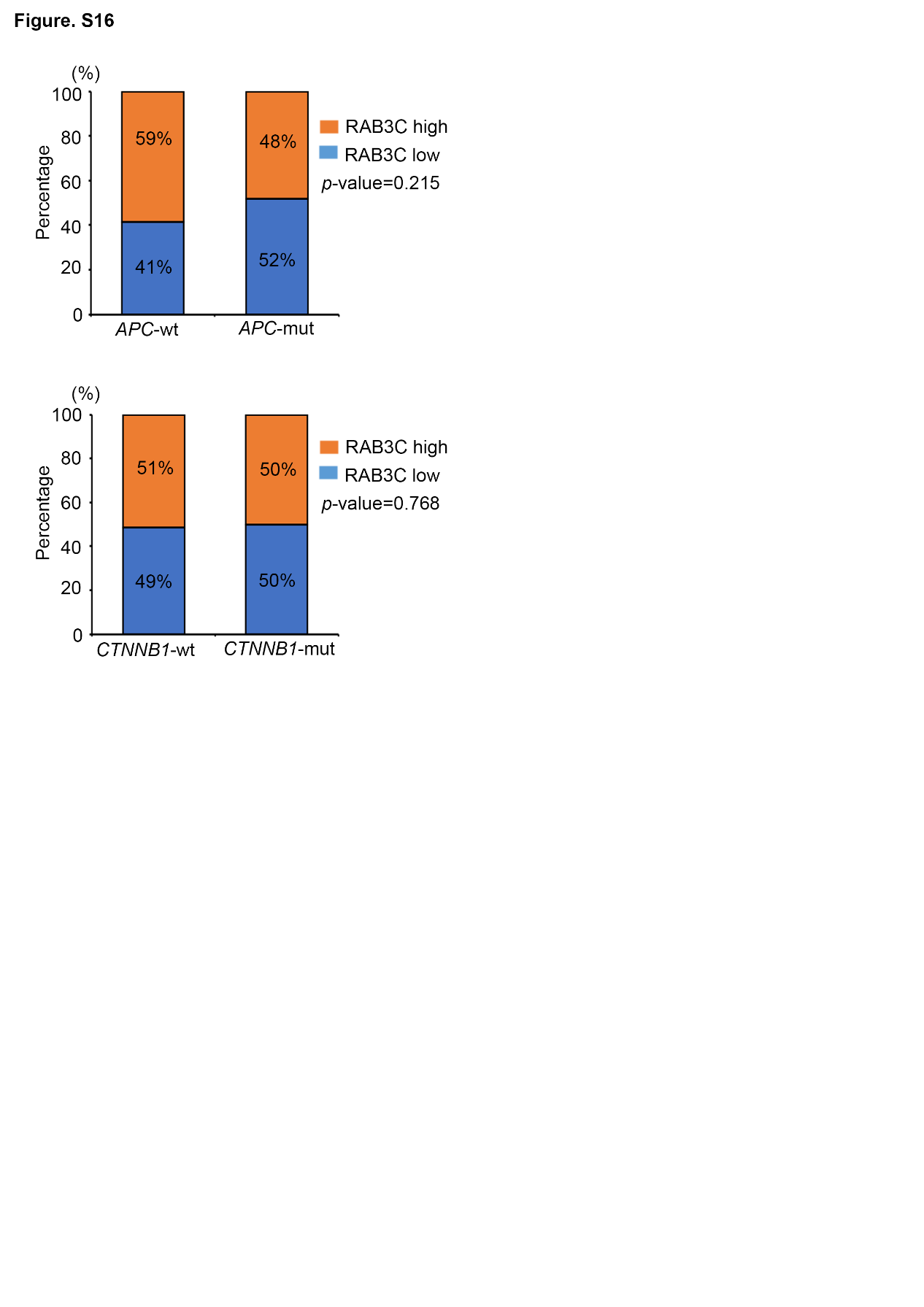
**

**Supplementary Figure S18.** The percentage of RAB3C expression levels in the wild-type and mutant *APC and CTNNB1* groups, respectively. wt: *The APC* or *CTNNB1* gene is wild-type. mut: *Mutation of the APC* or *CTNNB1 gene.*


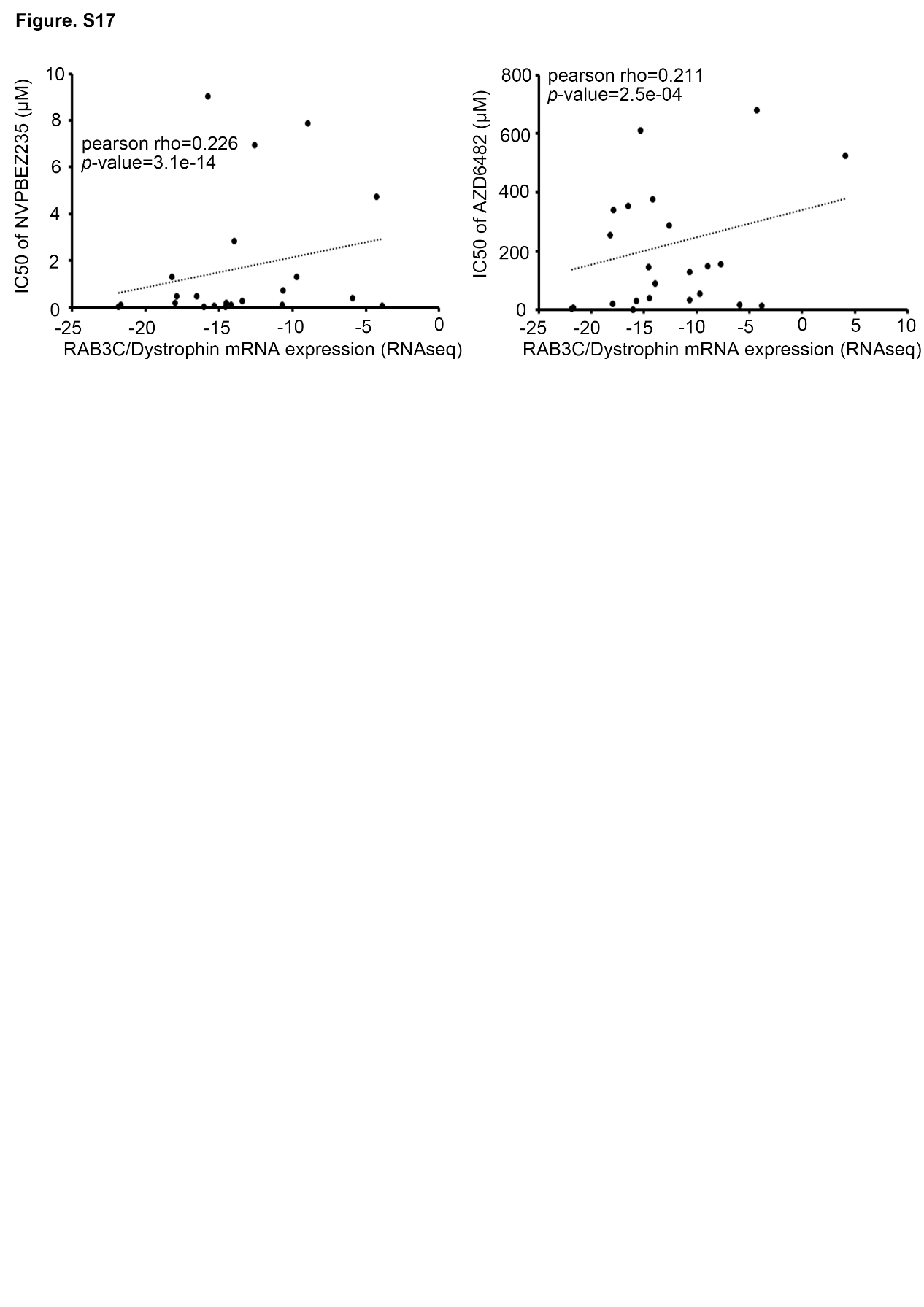


**Supplementary Figure S19.** The correlation plot of IC50 of NVPBEZ235 and AZD6482 with RAB3C/dystrophin mRNA expression level in colon cancer cells.
